# Differential roles of Na_V_1.2 and Na_V_1.6 in regulating neuronal excitability at febrile temperature and distinct contributions to febrile seizures

**DOI:** 10.1038/s41598-017-17344-8

**Published:** 2018-01-15

**Authors:** Mingyu Ye, Jun Yang, Cuiping Tian, Qiyu Zhu, Luping Yin, Shan Jiang, Mingpo Yang, Yousheng Shu

**Affiliations:** 1 State Key Laboratory of Cognitive Neuroscience and Learning, School of Brain and Cognitive Sciences, the Collaborative Innovation Center for Brain Science, Beijing Normal University, Beijing, China; 2 Institute of Neuroscience, State Key Laboratory of Neuroscience, Shanghai Institutes for Biological Sciences, Chinese Academy of Sciences, Shanghai, China; 3 iHuman Institute, ShanghaiTech University, Shanghai, China; 4 Brain Institute, College of Pharmaceutical Sciences, Capital Medical University, Beijing, China

## Abstract

Dysregulation of voltage-gated sodium channels (VGSCs) is associated with multiple clinical disorders, including febrile seizures (FS). The contribution of different sodium channel subtypes to environmentally triggered seizures is not well understood. Here we demonstrate that somatic and axonal sodium channels primarily mediated through Na_V_1.2 and Na_V_1.6 subtypes, respectively, behave differentially at FT, and might play distinct roles in FS generation. In contrast to sodium channels on the main axonal trunk, somatic ones are more resistant to inactivation and display significantly augmented currents, faster gating rates and kinetics of recovery from inactivation at FT, features that promote neuronal excitabilities. Pharmacological inhibition of Na_V_1.2 by Phrixotoxin-3 (PTx3) suppressed FT-induced neuronal hyperexcitability in brain slice, while up-regulation of Na_V_1.2 as in Na_V_1.6 knockout mice showed an opposite effect. Consistently, Na_V_1.6 knockout mice were more susceptible to FS, exhibiting much lower temperature threshold and shorter onset latency than wildtype mice. Neuron modeling further suggests that Na_V_1.2 is the major subtype mediating FT-induced neuronal hyperexcitability, and predicts potential outcomes of alterations in sodium channel subtype composition. Together, these data reveal a role of native Na_V_1.2 on neuronal excitability at FT and its important contribution to FS pathogenesis.

## Introduction

Febrile seizure (FS) that occurs during fever is a major convulsive form in pediatric population, affecting 2–5% children below age 6^1,2^. Both genetic and environmental factors may contribute to the pathogenesis of FS. Mutations in genes encoding sodium channels^3–6^, GABA_A_ receptors^7–9^ and interleukins^10^, have been implicated in conferring susceptibility to FS. On the other hand, hyperthermia or excess heat per se has been well documented to be sufficient to provoke seizures in experimental animals and clinical cases^2,11–14^. Due to the fact that temperature affects numerous molecular and cellular processes, many factors could contribute to FS pathogenesis. Indeed, a number of hypotheses had been proposed^2^. Among them the prevalent hypotheses are hyperventilation-induced alkalosis and cytokine release during fever. However, the former doesn’t well conform to some experimental FS models in which the subjects without experiencing hyperventilation also developed seizures^12^ and its relevance to human conditions remains to be established^15^. While the latter may promote the generation and exacerbation of FS^16^, the time scales for synthesis and release of IL-1b from several hours to days might not well temporally correlate with the acute onset nature of FS (typically within 30 min exposure to excess heat)^17,18^. Thus, other mechanisms should be involved. As FT is sufficient to induce FS in rodent pups without genetic defects^2,11–14^, we aimed to investigate the alternative potential underlying mechanisms of the environmentally triggered seizures.

VGSCs are fundamental molecules in determining neuronal excitability. A plethora of loss-of function(LoF) or gain-of-function(GoF) mutations of sodium channels have been identified in pedigrees with generalized epilepsy with febrile seizures plus (GEFS+), benign familial neonatal-infantile seizures (BFNIS), and severe myoclonic epilepsy of infancy (SMEI, or Dravet syndrome)^5,19,20^. LoF mutation in SCN1B (e.g.: p. C121W which causes slower inactivation of sodium currents without affecting recovery kinetics^3^) is linked to GEFS+ type 1; De novo LoF mutations in SCN1A which results in reduced currents specifically in interneurons leading to network dis-inhibition is associated with GEFS+ type 2, or Dravet syndrome^4,21^; Mutations in SCN2A (e.g. GoF mutation at p.Y1589C causes depolarizing shift of steady-state inactivation, slowed inactivation, increased persistent current and fasten recovery from inactivation^22^) have been associated with GEFS+, SMEI and BFNIS^5,22–25^; LoF mutations in SCN8A result in movement disorders and intellectual disability without seizures^26,27^, while GoF mutations in SCN8A (e.g. p. T767I mutation causes hyperpolorizing shift in the activation curves^28^) are associated with severe early-infantile epileptic encephalopathy type 13 (EIEE13)^29–32^.

VGSCs are also well known being sensitive to temperature changes^33–35^. Pathogenic LoF or GoF of sodium channels could also occur under hyperthermia, such as fever. Although it is evident that increased temperature plays a major role in FS pathogenesis, studies on the effect of FT on the gating mechanisms of sodium channel subtypes or their mutants associated with FS are largely lacking^36^. Thomas *et al*. showed in cell culture that FT causes GoF changes in Na_V_1.2 via hyperpolarization-shifting its V_50_ of activation^37^. Volkers *et al*. compared temperature effects on Na_V_1.1 wild-type, R859H, and R865G and showed LoF gating defects in both mutants at FT^38^; Peters *et al*. showed that the Dravet syndrome associated Na_V_1.1 mutant (p.A1273V) undergoes depolarization shifts in both steady state activation(LoF) and inactivation(GoF) at FT^39^. Studies in this direction could provide useful views on the mechanisms of FS at the molecular level, and thus could benefit rational development of treatments.

Sodium channel subtypes Na_V_1.2 and Na_V_1.6 are two predominant forms in excitatory pyramidal neurons in the cerebral cortex with localization specificity and may cooperate for the initiation and propagation of action potentials (APs)^40^. Little is known how they behave at febrile temperature (FT, 40–41 °C) and how that may contribute to seizures. In present study, we performed patch-clamp recordings at different temperature for somatic and axonal sodium channels directly isolated from *ex vivo* preparations. In combining with pharmacology, behavior assay and neuron simulation approaches, we investigated their contributions to neuronal excitability at FT and seizure susceptibility. Our data differentiate the temperature dependent biophysical properties of somatic and axonal sodium channels primarily Na_V_1.2 and Na_V_1.6 respectively, and reveal an important role of Na_V_1.2 subtype in supporting neuronal hyperactivities at FT that may induce seizures.

## Results

**Figure 1:**
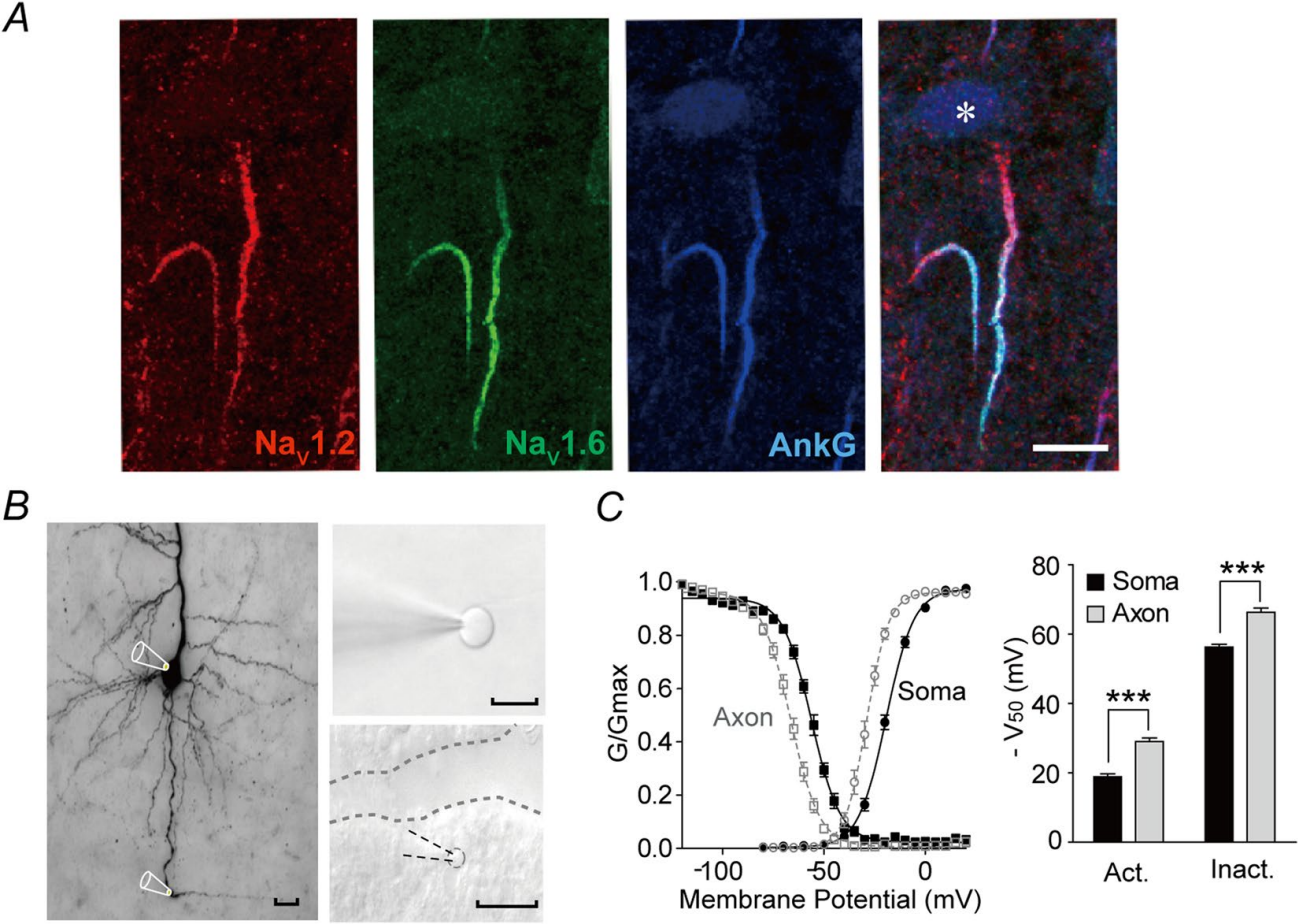
Different voltage-dependent properties of somatic and axonal sodium channels. (**A**) Triple staining for Na_V_1.2(red), Na_V_1.6(green), and Ankryn-G(blue) showing abundant proximal AIS localization of Na_V_1.2 versus dense distal AIS distribution of Na_V_1.6 subtypes on SD rat prefrontal cortical L5 pyramidal neurons. *Labels soma; Scale bar: 10 µm. (**B**) Left, illustration of recording configurations on a DAB-stained prefrontal cortical pyramidal neuron. Right, examples of nucleated patch (top) and isolated axonal bleb (bottom) recording. Dash lines contour patch pipette, axonal bleb and a cut through layer 6 and white matter to disconnect the bleb and the main axon. Scale bar: 15 µm. (**B**) Left, normalized activation and inactivation curves of somatic and axonal sodium channels, respectively. Right, comparison of V_50_s of activation and inactivation curves. ***p < 0.0001.

Previous electrophysiology and immunocytochemistry studies showed that Na_V_1.2 subtypes are expressed at the proximal axonal initial segment(AIS) and soma, while Na_V_1.6 subtypes distribute along distal AIS and axonal trunks of cortical pyramidal neurons^40,41^. We also performed immunostaining for Na_V_1.2 and Na_V_1.6 on prefrontal cortical tissues. In consistent to our previously published data, Na_V_1.2 channels are found abundantly expressed in AIS proximal to soma while Na_V_1.6 localize at distal AIS and axonal tracts of the layer 5 pyramidal neurons (Fig. 1A). Notably, Na_V_1.2 and Na_V_1.6 are the major sodium channel subtypes on excitatory neurons, while another neuronal specific subtype, Na_V_1.1, is only found on interneurons, e.g. PV positive one (Suppl. Figure 1) and Na_V_1.3 is undetectable on postnatal 14 brain tissues (Suppl. Figure 2), a result consistent with previous reports^19,41–44^.

To study the biophysical properties of different native sodium channel subtypes at various temperature conditions, we made patch clamp recordings on sodium currents from isolated somatic nucleated patches and axonal blebs (Fig. 1B). Consistent with previous data^40^, the V_50_s of somatic sodium channels’ activation/inactivation curves were approximately 10 mV more depolarized than those of axonal channels (two-way ANOVA: soma or axon location effect: F_(1,122)_ = 1211.2, p < 0.0001, Fig. 1C, Suppl. Table 1), suggesting that the sodium currents recorded from somatic nucleated patches and isolated axon blebs in our preparations are primarily derived from Na_V_1.2 and Na_V_1.6 channels, respectively.

### FT significantly enhances the functionality of somatic sodium channels but not axonal ones

**Figure 2:**
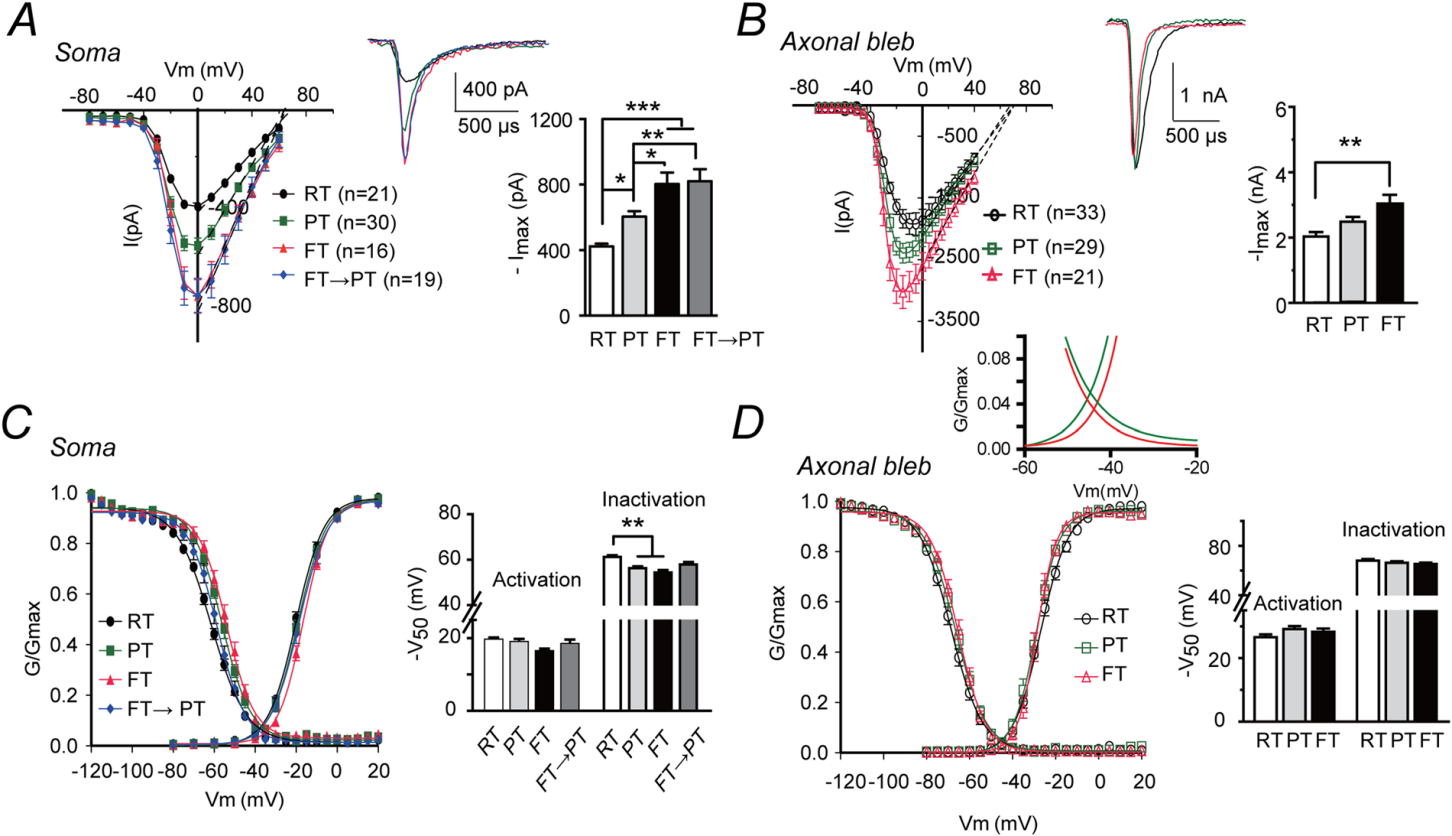
FT significantly enhances the somatic sodium currents. (**A**) Comparison of somatic sodium currents recorded at RT, PT and FT. Left: I-V curves; Right, bar graph of peak currents. Inset: representative sodium current traces at different temperatures. (**B**) similar as (**A**), but for axonal sodium channels. (**C**) Left: comparison of normalized activation and inactivation curves for somatic sodium channels at different temperatures. Right: bar graph comparison of V_50_s. (**D**) Similar to panel (**C**) but for axonal sodium channels. Inset: zoom in view for comparing the areas under curves (window currents) between PT (green Boltzmann fitting curve) and FT (red Boltzmann fitting curve). *p < 0.05; **p < 0.01; ***p < 0.0001. Black traces, RT; Green, PT; Red, FT; Blue: FT → PT.

We then examined the temperature responsive properties of Na_V_1.2 and Na_V_1.6 sodium channel subtypes directly excised from the somatic and axonal compartments of cortical pyramidal cells, respectively. We first compared the current amplitudes at different temperatures because this parameter reflects the net result from changes in open probability and conductance at single channel level^45^. Larger current amplitudes suggest more channels opening or larger conductance mediated through single channels, thus enhanced neuronal excitability. Our data showed that the peak amplitude of somatic sodium currents recorded at FT(n = 16) was 90.3% and 33.0% larger than those recorded at room temperature(RT, 25 °C, n = 21) and physiological temperature(PT, 36 °C n = 30), respectively (One-way ANOVA: F_(3,82)_ = 13.6; post-hoc Bonferroni test, p < 0.05 between PT and FT; Q_10(PT->FT)_ of 2.04; Fig. 2A, Suppl. Figure 3 for example traces, Suppl. Table 1). This effect was not readily reversible. After 30-min incubation at FT followed by over 10 min cooling to PT (n = 19), the sodium currents remained comparatively large as those at FT (p > 0.05, between FT and FT → PT; p < 0.01 between PT and FT → PT, Fig. 2A). In contrast, the amplitudes of axonal sodium currents did not change significantly between PT (n = 29) and FT (n = 21), except for comparing with those at RT (n = 33; One-way ANOVA: F_(2,80)_ = 6.4, post-hoc Bonferroni test, p > 0.05 between PT and FT; p < 0.005 between RT and FT or RT and PT, Fig. 2B, Suppl. Table 1–3). Notably, inconsistent with the previous *in vitro* study^37^, the normalized G-V curves of both somatic and axonal sodium channels were stable. No significant difference was found in the V_50_s and slope factors between PT and FT(One-way ANOVA: p > 0.05; Fig. 2C,D, Suppl. Table 1–3), except for the slope factor of activation curves of axonal channels(p < 0.05 between PT and FT, One way ANOVA, Suppl. Table 1–3). Smaller slope factor (a steeper G-V curve) at FT of axonal currents indicates less activation of axonal sodium channels at voltages negative to V_50_, which results in smaller window currents thus fewer channels that would be activated but not fully inactivated at FT (Fig. 2D, inset). Besides, FT caused ~2 mV depolarizing, instead of hyperpolarizing^37^, shifts in the V_50_s of somatic channels. The discrepancy could be attributed to the fact that, unlike *in-vitro* expression of sodium channel subunits, the sodium channels’ integrity as well as their local cellular environment was largely preserved in our preparations through *in situ* isolation of cellular compartments of neurons in brain slices.

**Figure 3:**
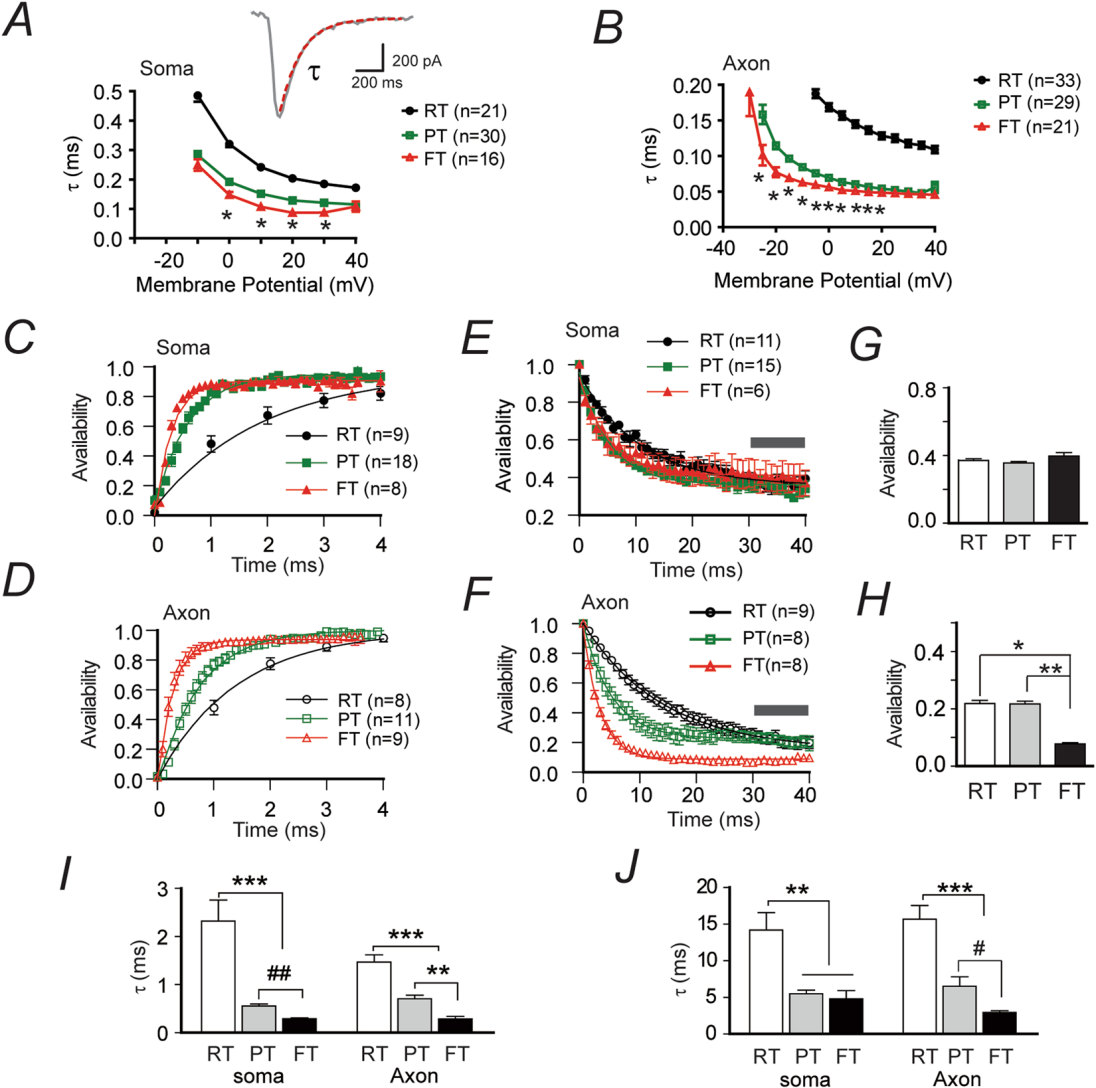
Faster sodium channel gating rates and kinetics at higher temperature. (**A**) Plot of decay time constants of somatic sodium currents at different temperatures across a range of membrane potentials from −30 mV to 40 mV. Inset: a representative current trace and its exponential fit. Significant difference between PT and FT were found from 0 to 30 mV steps. *:at least p < 0.05, unpaired t-test. Curve for data at PT → FT was not shown for comparison clarity between PT and FT, but example data is presented in suppl. Table 1-2. (**B**) Similar to panel (**A**), but for axonal sodium channels. Significant difference between PT and FT was found from −40 to 20 mV step. *:at least p < 0.05, unpaired t-test. (**C**,**D**) Kinetics of recovery from inactivation for somatic (**C**) and axonal (**D**) sodium channels at different temperatures. (**E**,**F**) Kinetics of onset inactivation for somatic (**E**) and axonal (**F**) sodium channels at different temperatures. Gray bars indicate the sustained currents after prolonged depolarization which were used for comparison shown in (**G**) and (**H**). (**I**,**J**) Bar graph comparison of time constants for the kinetics of recovery from inactivation (I) and the kinetics of onset inactivation (**J**) for somatic and axonal sodium channels. *p < 0.05; **p < 0.01; ***p < 0.0001, One-way ANOVA with post-hoc Bonferroni’s multiple comparison. #p < 0.05, ##p < 0.005, unpaired t-test.

We next measured the sodium channel gating rates, onset inactivation and recovery kinetics at various temperatures as these parameters are known to be critical in affecting neuronal excitability. Our data show that both somatic and axonal sodium channels exhibited faster gating rates at higher temperature as exemplified by reduced decay time constants of the sodium currents (Fig. 3A,B). The recovery from inactivation of both channels also developed faster at higher temperature (Fig. 3C,D,I, Suppl. Table 1–3, and Suppl. Figure 4 for example traces). Notably, the time constants for the development of inactivation did not alter much between PT (n = 15) and FT (n = 6) for the somatic sodium channels (p > 0.05, unpaired t-test, Fig. 3E,J), but were significantly reduced for the axonal ones (p < 0.01 between PT (n = 8) and FT (n = 8), unpaired t-test, Fig. 3F, and J, example traces shown in Suppl. Figure 4). Besides, the sustained somatic sodium currents after prolonged depolarization at FT did not decrease significantly as compared to those at RT and PT (p > 0.05, between PT and FT, F_(2,27)_ = 0.44; One-way ANOVA, Fig. 3G). In contrast, the availability of axonal sodium currents rapidly reduced at FT within 40-ms depolarization period (p < 0.01 between PT and FT, F_(2,16)_ = 7.6, One-way ANOVA, Fig. 3H). These data indicate that the axonal sodium channels inactivate faster and more extensively than the somatic ones at FT, suggesting the axonal ones might exert limited roles in supporting neuronal firing at FT due to their massive inactivation.

Together, the difference in biophysical behaviors of somatic channels (primarily Na_V_1.2) and axonal sodium ones (mainly Na_V_1.6) at FT strongly suggest their distinct contributions to neuronal excitability.

#### FT promotes neuronal excitability

**Figure 4:**
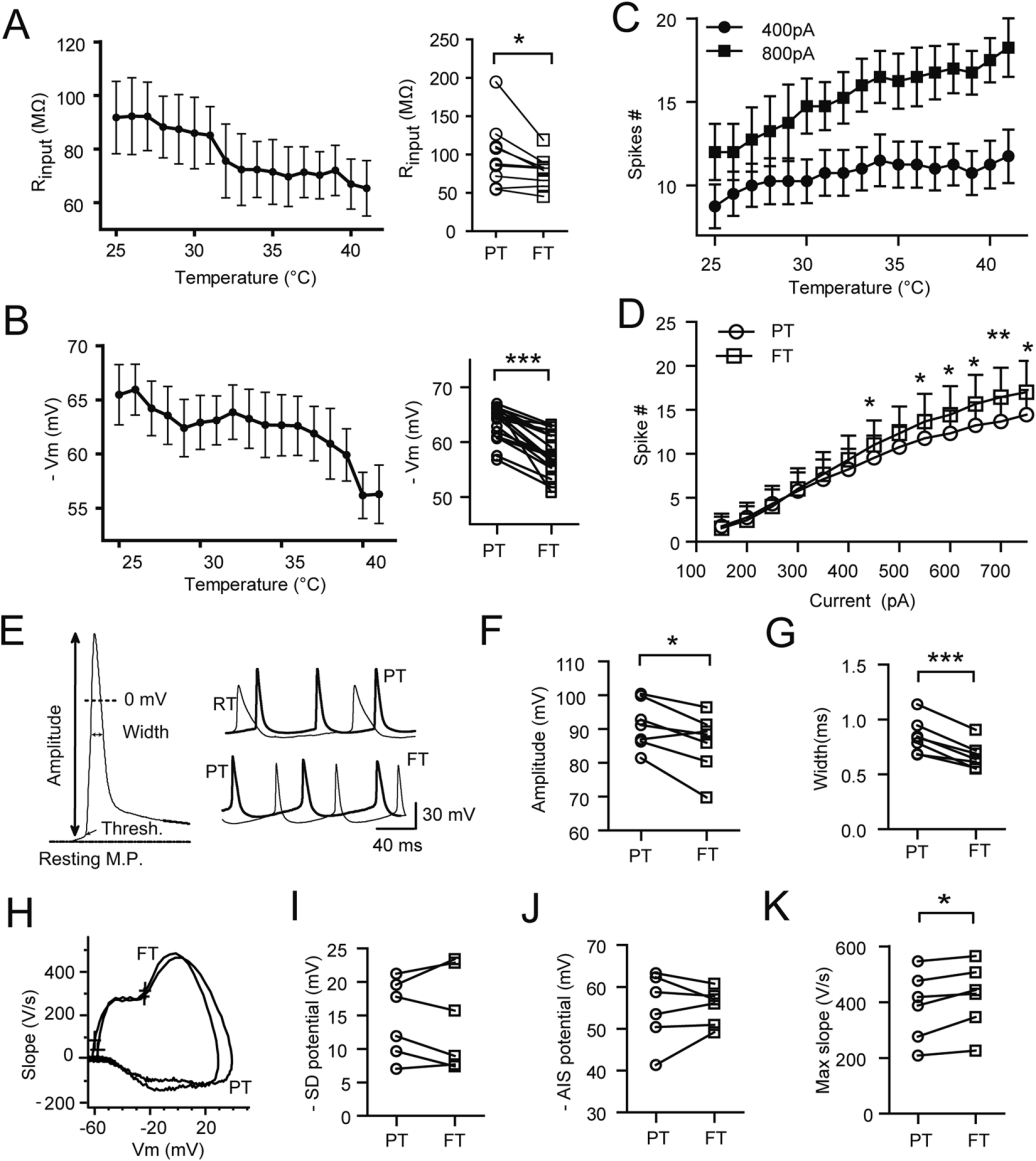
FT promotes neuronal excitability. (**A**) Left: input resistance continuously reduced as temperature increased from RT to FT, Right: paired comparison of Rinput between PT and FT. (**B**) similar to (**A**) but for comparing the temperature effect on membrane potentials. (**C**) Temperature elevation promoted AP firing as measured at 400pA, 800pA current steps. (**D**) Enhanced neuronal excitability at FT measured at multiple current injection steps. (**E**) Left: an illustration on the measurement of AP parameters. Right: Overlap of AP waveforms at RT and PT, PT and FT. (**F**,**G**) Paired comparison of the width and amplitudes of APs at PT and FT. (**H**) an example of phase plot of backpropagated APs recorded at PT and FT. Crosses marked the AIS and SD threshold potentials. Paired comparison of SD (**I**) and AIS(**J**) threshold potentials between PT and FT. (**K**) Paired comparison of the maximum slope of backpropagated APs between PT and FT. *p < 0.05; **p < 0.01; ***p < 0.001. Paired t test.

Next we asked how increasing temperature from PT to FT might affect neuronal intrinsic properties and excitability. To this end, we performed whole-cell current clamp recordings at the soma with ramp temperature increases and fixed steps of current injections or with a series of current injections at fixed steps of temperature. Temperature ramp-up from RT to FT caused a progressive decrease in input resistance (Rin) and depolarized membrane potentials (Vm; n = 4; Fig. 4A left, 4B left). Group data showed FT caused 21% reduction in Rin from 99.4 ± 14.4 MΩ at PT to 78.5 ± 6.9 MΩ at FT (n = 9; p < 0.05; Fig. 4A right), and a depolarization from −63.5 ± 0.7 mV to −58.1 ± 0.9 mV (n = 15, p < 0.0001; paired t-test, Fig. 4B right). FT enhanced neuronal excitability as indicated by increased discharge of APs (APs; Fig. 4C, n = 4; 4D, n = 9). Comparison of AP waveforms revealed that FT shortened AP width by 20.5% (p < 0.01) and decreased AP amplitudes by 6.1% (p < 0.05; n = 7; paired t-test, Fig. 4E–G), suggesting that FT play a composite effect on the ion channels activated during APs. Together, these data demonstrate that FT directly affects neuronal intrinsic properties and promotes excitability.

Alterations in the threshold for AP initiation and the threshold of back-propagated APs to invade soma and dendrites may directly affect neuronal excitability. To investigate whether FT may affect AP initiation and back-propagation, we made dual current clamp recordings at the soma and the axon bleb simultaneously and recorded action potentials back-propagating from axonal bleb to soma. The thresholds of both axon initial segment (AIS) potential and somatodendritic(SD) potential^40^ at PT and FT did not alter significantly (paired t-test, p > 0.05; n = 7, Fig. 4H–J). Notably, the maximal slopes (dV/dt) of action potentials at FT increased by 5.1% comparing with those at PT (paired t-test, p < 0.05; n = 7; Fig. 4K), suggesting an enhancement of sodium currents (dV/dt = I/C) at FT which is consistent with previous direct measurement.

### Na_V_1.2 channels mediate febrile temperature induced enhancement of neuronal excitability

Based on our previous electrophysiology data, increased expression of Na_V_1.2 channels would be expected to facilitate neuronal excitability at FT, while blocking them would have an opposite effect. To test this hypothesis, we used Na_V_1.6 homozygous knockout mice (*Scn8a*^*−/−*^), in which Na_V_1.2 channels were found to be compensatorily upregulated^46–48^. On the other hand, due to Na_V_1.2 knockout is perinatal lethal^49^, we chose pharmacological approaches, using a Na_V_1.2 specific blocker, PTx3, to isolate Na_V_1.6 currents in brain slices.

**Figure 5:**
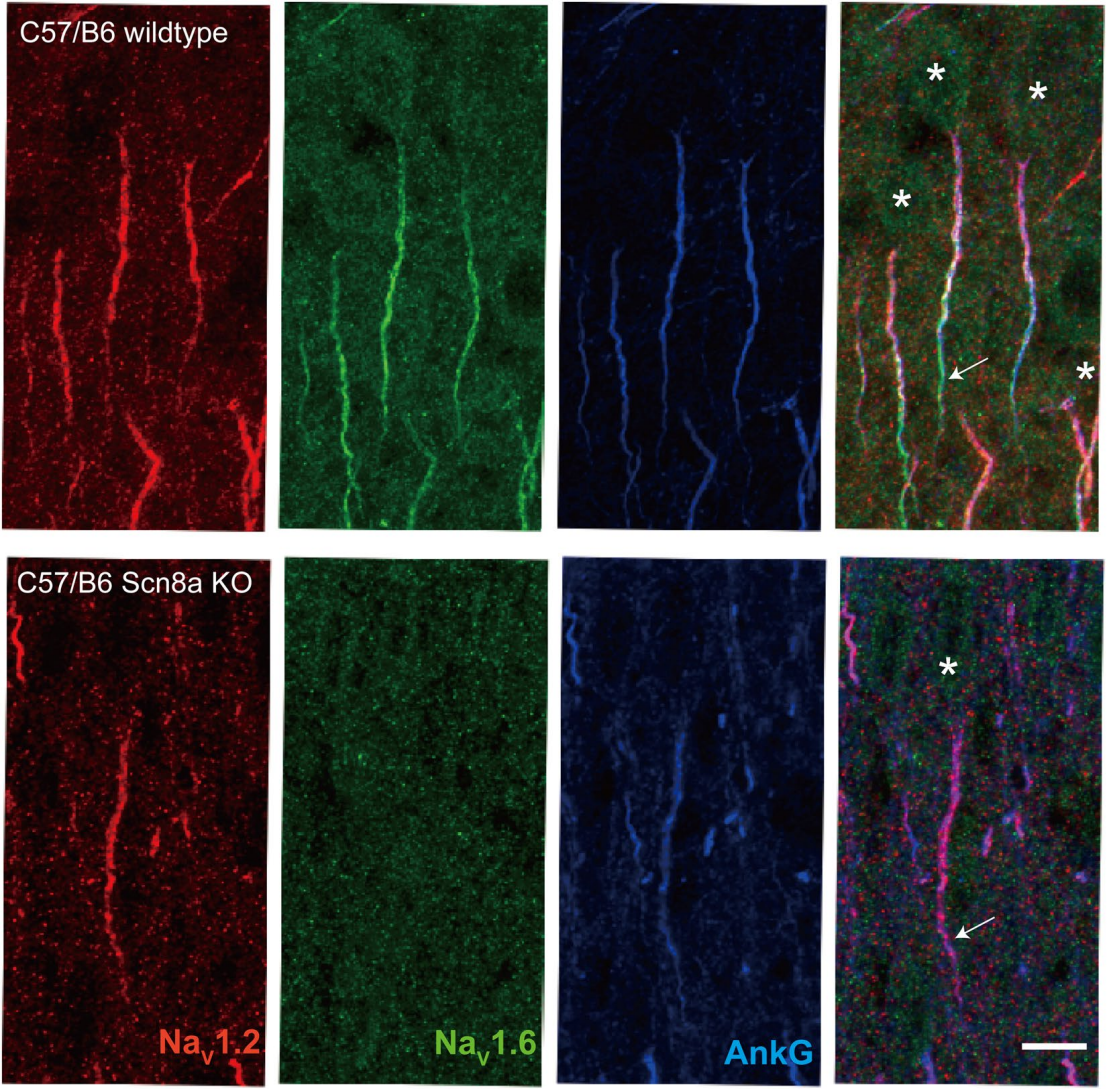
Compensatory expression of Na_V_1.2 on pyramidal neurons of *Scn8a*^*−/−*^ mice. (**A**) Triple staining of Na_V_1.2 (red), Na_V_1.6(green) and AnkG (blue) on wildtype C57/B6 brain cortical tissue shows stereotypic expression pattern of Na_V_1.2 and Na_V_1.6. Arrow labels distal AIS with Na_V_1.6 positive staining. (**B**) Similar as (**A**) but from cortical sections of *Scn8a*^*−/−*^ mice. Note that Na_V_1.6 is undetectable and replaced with Na_V_1.2. Arrow indicates Na_V_1.2 immunopositive staining at distal AIS. *Labels soma position. Scale bar: 10 µm.

We performed immunostaining for Na_V_1.2 and Na_V_1.6 channels on prefrontal cortical tissues of both wildtype and *Scn8a*^*−/−*^ mice. As shown in Fig. 5 top panel, Na_V_1.2 and Na_V_1.6 have a spatial expression pattern on the pyramidal neurons of wildtype mice similar to that of SD rats (Fig. 1A). Importantly, in *Scn8a*^*−/−*^ mice, Na_V_1.6 is undetectable and is compensatorily replaced by Na_V_1.2 on the distal AIS (Fig. 5 bottom panel).

**Figure 6:**
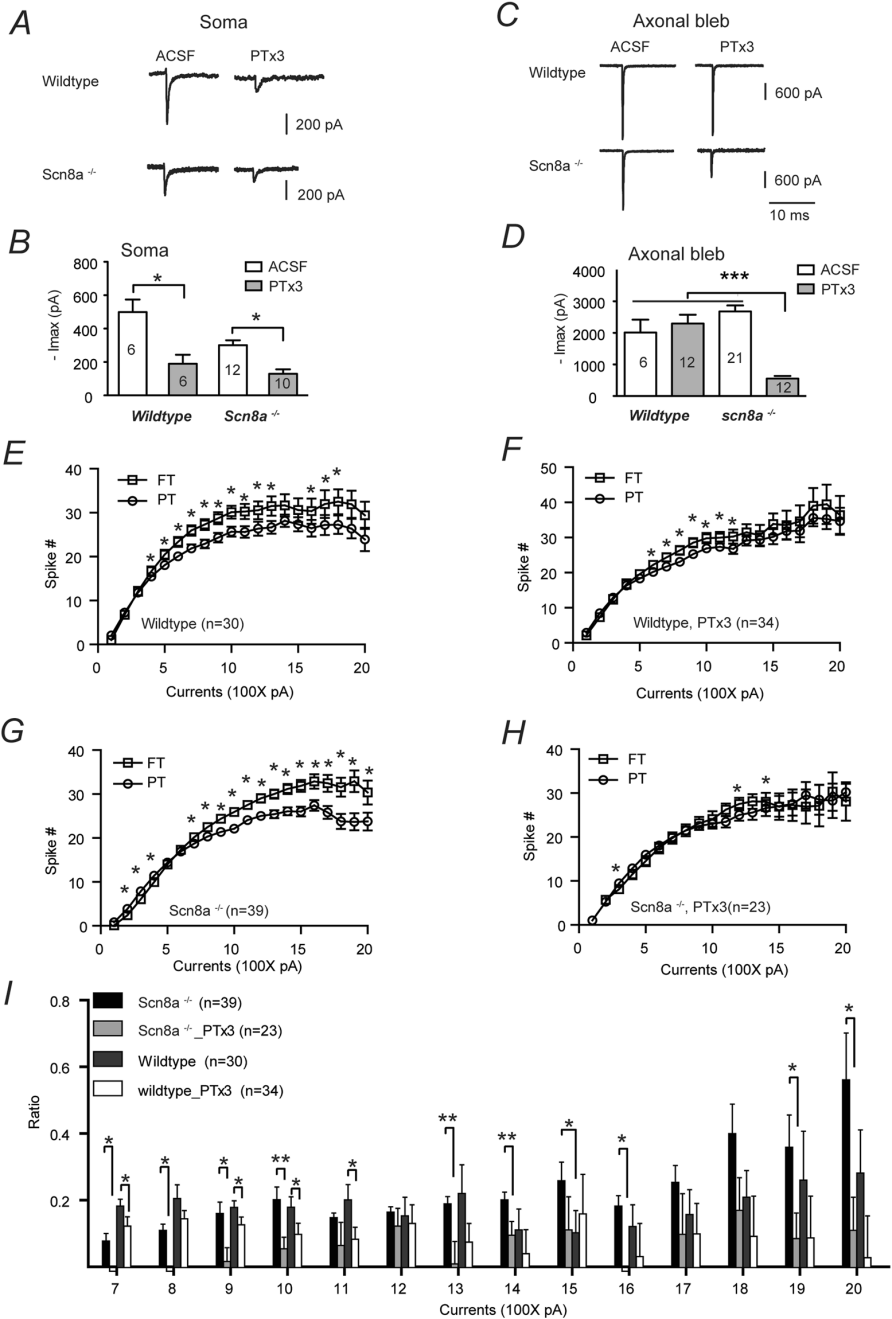
Blockade of Na_V_1.2 suppresses FT-induced enhancement of excitability in *Scn8a*^*+/+*^ and *Scn8a*^*−/−*^ neurons. (**A**) Example traces showing that bath application of PTx3 effectively suppresses somatic sodium currents from both wildtype and *Scn8a*^*−/−*^ neurons. (**B**) Comparison of somatic sodium currents of wildtype and *Scn8a*^*−/−*^ neurons with and without the presence of PTx3. (**C**) Example traces showing that PTx3 suppresses axonal sodium currents of *Scn8a*^*−/−*^, but not wildtype neurons. (**D**) Similar as (**B**) but for axonal sodium currents. Number of neurons is shown in the bars. *p < 0.05; **p < 0.01. One-way ANOVA with Bonferroni’s multiple comparisons. (**E**–**H**): Bath application of PTx3 suppressed FT induced enhancement of AP firing in both wildtype (**E**: without PTx3; F: with PTx3) and *Scn8a*^*−/−*^ neurons (**G**: without PTx3; **H**: with PTx3). Paired t-test. (**I**) Bar graph showed the blockade of Na_V_1.2 by PTx3 results in significant reduction in the excitability-promoting effects of FT across a number of current injection steps in both wildtype and *Scn8a*^*−/−*^ neurons. *p < 0.05; **p < 0.01; unpaired t-test.

We also verified that Na_V_1.2 channels were indeed upregulated in pyramidal neurons of *Scn8a*^*−/−*^ mice via direct electrophysiological measurement. Somatic nucleated patches recordings showed that bath application of 180 nM Phrixotoxin-3 (PTx3) effectively blocked somatic sodium currents from neurons of both wildtype and *Scn8a*^*−/−*^ mice (Fig. 6A,B. F_(3,30)_ = 13.1, One-way ANOVA, p < 0.0001), suggesting the current was primarily derived from Na_V_1.2 channels. Differently, isolated axonal bleb recordings showed that PTx3 only effectively blocked the axonal sodium currents from neurons of *Scn8a*^*−/−*^, but not wildtype mice (Fig. 6C,D. F_(3,47)_ = 18.0, One-way ANOVA, p < 0.0001), suggesting a functional replacement of mutated Na_V_1.6 channels with normal Na_V_1.2 on the axonal trunks of *Scn8a*^*−/−*^ neurons. Thus, our electrophysiology data also support that Na_V_1.2 channels were compensatorily upregulated in pyramidal neurons of *Scn8a*^*−/−*^ mice.

Next we evaluated the effect of temperature on neuronal excitability by recording *Scn8a*^*−/−*^ and wildtype neurons with or without bath application of Na_V_1.2 blocker. Both wildtype (n = 30, Fig. 6E) and *Scn8a*^*−/−*^ (n = 39, Fig. 6F) neurons displayed enhanced excitability at FT. Importantly, the FT-induced increase in excitability could be significantly suppressed by PTx3 measured over multiple current injection steps (e.g. 700pA to 1.1 nA 1.3–1.6 nA, 1.9–2.0 nA steps) for both wildtype and *Scn8a*^*−/−*^ neurons (Fig. 6F,H,I, Suppl. Table 4), suggesting that Na_V_1.2 is required in mediating FT-induced enhancement of neuronal excitability. Besides, the excitability enhancement effect was larger in *Scn8a*^*−/−*^ neurons than wildtype counterparts with significant difference found at 1500 pA current injection step (p < 0.05, unpaired t-test, Fig. 6I), suggesting that upregulation of Na_V_1.2 in *Scn8a*^*−/−*^ neurons could promote FT induced neuronal excitability.

### ***Scn8a***^*−/−*^**mice are susceptible to febrile seizures**

**Figure 7:**
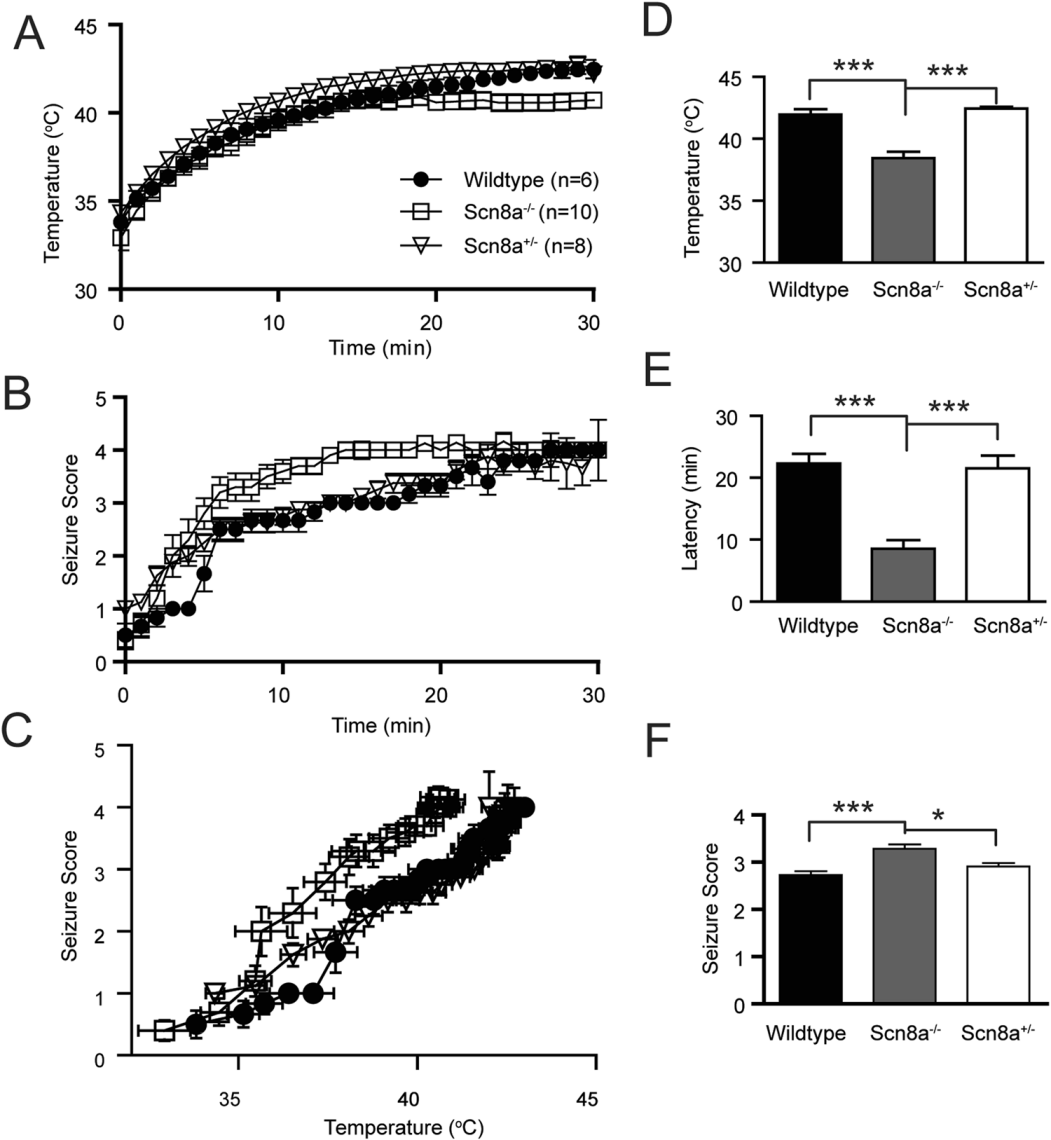
Enhanced susceptibility of *Scn8a*^*−/−*^ mice to FS. (**A**) Body temperature developing curves of *Scn8a*^*−/−*^ (n = 10)*, Scn8a*^+/−^ (n = 8), and wildtype (n = 6) mice. (**B**) Plot of seizure scores over the entire 30 min behavioral paradigm. (**C**) Plot of seizure scores versus body temperature. (**D**–**F**) Comparison of seizure threshold temperature (**D**), onset latency (E), and severity (**F**). *p < 0.05; ***p < 0.001. One-way ANOVA with post-hoc Bonferroni’s multiple comparisons.

Previous studies showed that *Scn8a*^+/−^ mice were resistant to seizures induced by chemicoconvulsants, such as flurothyl and kainite acid^26^. It is unknown whether *Scn8a*^*−/−*^ mice behave similarly following exposure to other forms of seizure inducers, such as hyperthermia. We hypothesized that upregulation of Na_V_1.2 in *Scn8a*^*−/−*^ mice could predispose the mice to FT-induced seizures. We thus accessed the FS susceptibility of mice with different genetic backgrounds. Briefly, wildtype (*Scn8a*^+/+^), knockout (*Scn8a*^*−/−*^), and heterozygous knockout (*Scn8a*^+/−^). Littermates were individually subjected to an isolated environment pre-warmed at 42 °C. Seizure severity, latency, ambient and body temperature were monitored and assessed. As shown in Fig. 7A, the core temperature of all tested mice developed in good consistency, suggesting a comparable homeostatic function on thermal regulation. Notably, knockout mice were more prone to febrile seizures (Fig. 7B and C) with a threshold temperature ~4 °C lower than *Scn8a*^*+/+*^ and *Scn8a*^+/−^ mice (respectively: 38.4 ± 0.5 °C, n = 10; 42.0 ± 0.4 °C, n = 8; 42.4 ± 0.1 °C, n = 6; F_(2,21)_ = 28.8, p < 0.0001, One-way ANOVA, Fig. 7D), and approximately 14 min shorter onset latency (respectively: 8.5 ± 1.4 min for *Scn8a*^*−/−*^; 22.3 ± 1.5 min, wildtype; 21.5 ± 2.1 min, *Scn8a*^+/−^. F_(2,21)_ = 22.5, p < 0.0001, One-way ANOVA, Fig. 7E). The integrated seizure score for *Scn8a*^*−/−*^ mice over the entire course was 13% and 20% higher than that for *Scn8a*^+/−^ and *Scn8a*^*+/+*^ mice respectively (F_(2,21)_ = 10.3, p < 0.0001, One-way ANOVA, Fig. 7F). Together, these data suggest that Na_V_1.2 could be a critical factor in conferring mice susceptibility to febrile seizures.

### Simulations reveal weighted contributions of Na_V_1.2 and Na_V_1.6 to neuronal excitability at FT

**Figure 8:**
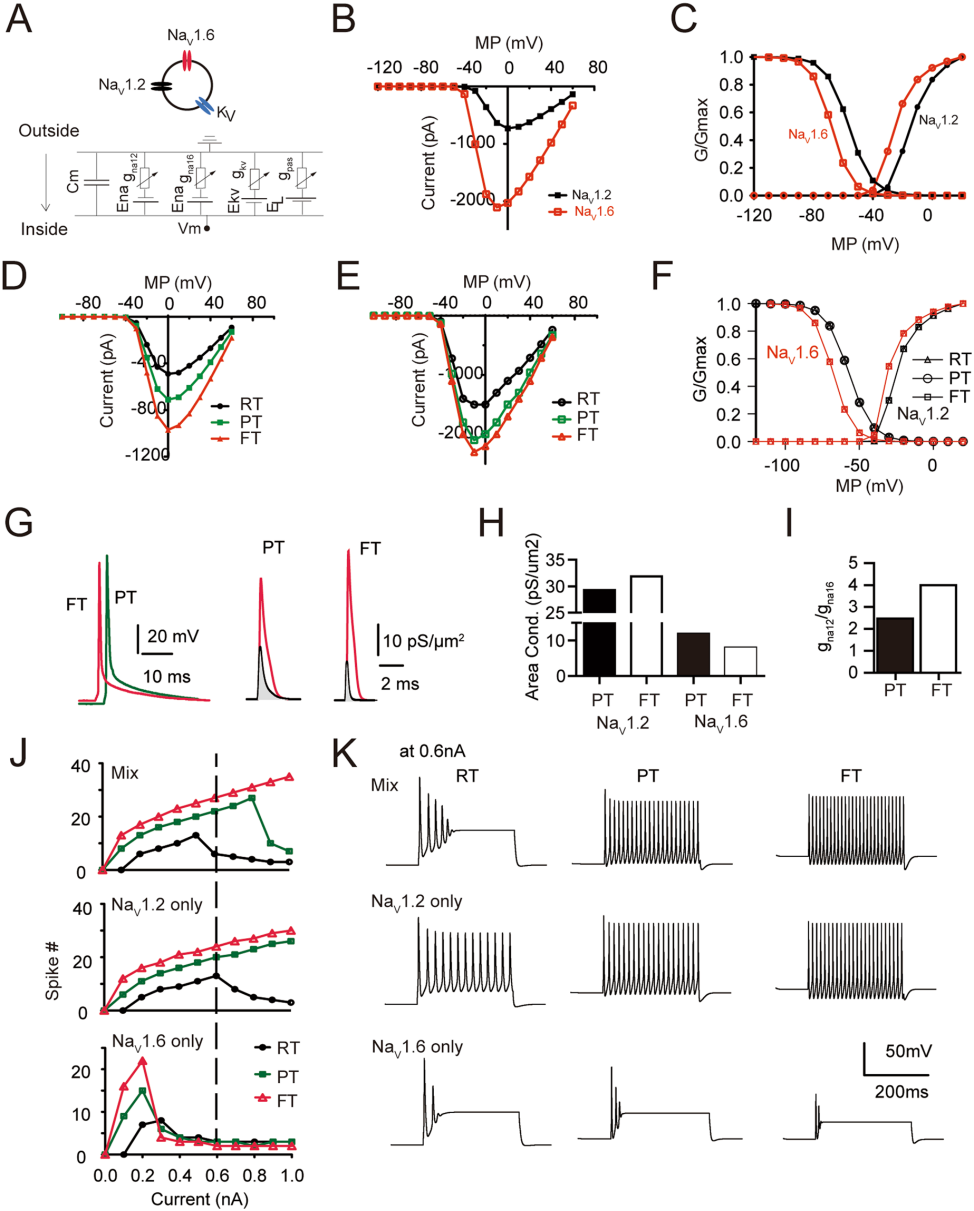
Simulations in a single compartment model. (**A**) Top: schematic illustration of the single compartment model. Bottom: equivalent electric model of the model neuron. (**B**–**F**) Adjustment of ion channel gating parameters and densities such that I-V, G-V curves and temperature effects on Na_V_1.2 and Na_V_1.6 channels mimics experimental observations. (**G**) Left: Overlap of example action potentials recorded at PT(green) and FT(red). Right:conductance of Na_V_1.2(red) and Na_V_1.6(black) underlying action potentials recorded at indicated temperatures. (**H**,**I**) Elevated temperature increased Na_V_1.2 conductance but reduced Na_V_1.6 conductance(H), resulting in an increased ratio of the conductance of Na_V_1.2 to Na_V_1.6(I). (**J**) Simulations on the role of different sodium channel subtypes on neuronal excitability at different temperature. (**K**) Example traces of APs from the model neuron fired at different temperatures and with different composition of sodium channel subtypes at a current injection step of 0.6 nA. Note that Na_V_1.6 subtype alone fails to support robust firing of model neurons.

To our knowledge, although there is a tarantula toxin derived Na_V_1.2-specific blocker PTx3, no specific blockers for Na_V_1.6 are available thus far. Conversely, although Na_V_1.6 null mice (*Scn8a*^*−/−*^) may survive for up to postnatal 3–4 weeks allowing for experimental assessment^26^, Na_V_1.2 knockout is perinatal lethal^49^. These limitations precluded a complete interrogation on the contributions of both sodium channel subtypes toward FS genesis. We therefore applied computational simulations to address these questions by constructing a single compartment model and a realistic neuron model. We adjusted the channel density and gating mechanism parameters in the single compartment model to approximate experimental observations on the properties of sodium channel subtypes (Fig. 8A–C for simulations of I-V curves, normalized G-V curves, Suppl. Figure 5A–D for simulations of channel gating time constants: τ_h_, τ_m,_ the kinetics of recovery from inactivation and development of inactivation), and also justified that our model recapitulated experimental observations on temperature effects on the channel gating time constants, kinetics of recovery from inactivation and development of inactivation (Fig. 8D–F, Suppl. Figure 5E–L).

We first analyzed the composition of sodium conductance underlying APs experimentally recorded at PT and FT from a pyramidal cell (Fig. 8G). Figure 8H showed that the integrated conductance of Na_V_1.2 channels underlying APs increased while Na_V_1.6 conductance decreased at FT in comparison with those at PT. The ratio of integrated Na_V_1.2 conductance over that of Na_V_1.6 was enhanced from 2.8 at PT to 4.0 at FT (Fig. 8I), suggesting that Na_V_1.2 channels provide a major sodium conductance supporting APs at FT. We further investigated the consequence of alterations in sodium channel subtype composition on neuronal excitability at various temperatures. Replacing all Na_V_1.2 with Na_V_1.6 (i.e. Na_V_1.6 only) caused failures of the model neuron in firing APs at FT, while substituting Na_V_1.6 with Na_V_1.2 (Na_V_1.2 only) allowed APs to be reliably generated at various tested temperature levels and step currents (Fig. 8J,K). Notably, the coexistence of Na_V_1.2 and Na_V_1.6 generated the highest firing rate of APs at FT, suggesting a synergistic effect of the two sodium channel subtypes. Together, these simulations indicated that Na_V_1.2 is instrumental in supporting neuronal excitability at FT.

**Figure 9:**
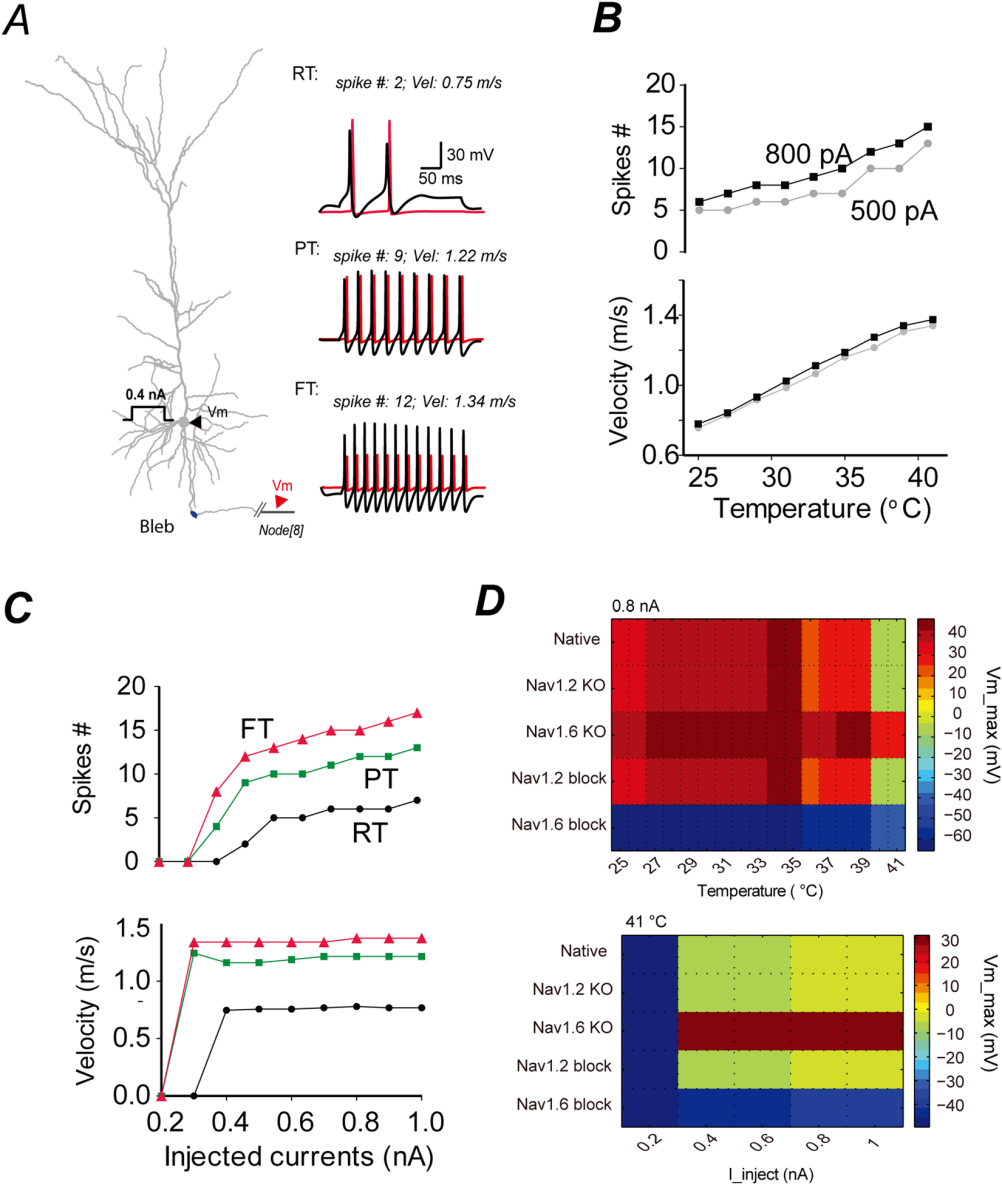
Simulations in a realistic neuron model. (**A**) Left, the morphology of a typical pyramidal neuron visualized with DAB staining was reconstructed for building realistic model. Black arrow head indicates the soma. Red arrow head indicates the 8th node of Ranvier 1306.5 µm away from the soma. Right, firing pattern and AP velocity of the model neuron at different temperatures at 0.4 nA current injection at the soma. Black traces: somatic APs. Red traces: axonal APs at the 8th node of Ranvier. (**B**) Effect of temperature on neuronal firing rate (upper) and AP conduction velocity (bottom). (**C**) Effect of current intensity on firing rate (upper) and AP conduction velocity (bottom) measured at RT (black), PT (green), and FT (red). (**D**) Simulation of the maximum depolarization level of spikes at the 8th Ranvier node in response to temperature elevation from 25 °C to 41 °C at 0.8 nA current injection step (Top), or in response to a series of current intensities at FT (Bottom). Note that Na_V_1.6 KO resulted in the highest depolarization level at the distal axon; Blockade of Na_V_1.6 effectively abolishes AP conduction along axons.

In realistic neuron model, we applied a 3D reconstructed neuron morphology of an experimentally recorded pyramidal neuron (Fig. 9A). The neuron model displayed increased firing rates and faster AP conduction velocity at higher temperature, indicating enhanced excitability (Fig. 9B,C and Suppl. Figure 6). In this model, the firing rates of the model neurons with different sodium channel subtype compositions don’t vary much at the same temperature (Suppl. Figure 6). However we noted that the upstrokes of action potentials propagating to the distal Ranvier nodes (e.g. the 8^th^ nodes) are the highest in the situation of Na_V_1.6 knockout with a compensatory substitution of Na_V_1.2. Higher level of depolarization would facilitate neurotransmitter release and thus increase post-synaptic excitability. On the other hand, blockage of Na_V_1.6 effectively abolished orthodromic propagation of action potentials along the axonal trunks (Fig. 9D). Together, these simulations reveal an important role of Na_V_1.2 subtype in supporting neuronal excitability at FT, and predict an effective way for the control of FS by instead targeting Na_V_1.6 to suppress the propagation of neuronal excitability.

## Discussion

How FT may trigger seizures at the molecular level in rodent pups without genetic defects is not well understood. In addition to the proposed hypotheses^2^, one possible route would be the acute effect of elevated temperature on ion channels or receptors leading to hyper-excitability at FT. Sodium channels are the fundamental molecules in determining neuronal excitability and are known to be sensitive to temperature changes. Little is known how they behave at FT and how that may contribute to FS genesis. Through direct isolation of different neuronal compartments by different patch clamp recording configurations, we studied the temperature responsive properties of somatic and axonal sodium channels primarily mediated through two sodium channel subtypes, Na_V_1.2 and Na_V_1.6, respectively, in excitatory layer 5 pyramidal neurons of rodent prefrontal cortex. Combining with pharmacological approaches, behavioral assay as well as computational modeling, our data demonstrate that FT differentially affects biophysical properties of the two channel subtypes, and its enhancement of neuronal excitability may be primarily mediated by Na_V_1.2 channels. Our data also show that FT alone is sufficient and efficacious to acutely provoke seizures in experimental mice in the absence of infection, inflammation and without genetic defects, a phenomenon in consistence with previous reports^12–14^. These data emphasize the importance of an environmental factor (i.e. febrile temperature) in FS pathogenesis, and reveal that native Na_V_1.2 channel could be a key player implicated in temperature responsive neuronal excitability and FT-induced seizures.

Through electrophysiological recordings we showed that FT acutely and directly enhanced the functions of somatic sodium channels, mainly Na_V_1.2 subtype. These included significantly augmented currents (Fig. 2A), faster gating rates (Fig. 3A) and kinetics of recovery from inactivation (Fig. 3C,3I). The axonal channels, primarily Nav1.6 subtype, also displayed faster gating rates (Fig. 3B) and recovery from inactivation (Fig. 3D,3I), but did not show significant enhancement in the currents at FT (Fig. 2B). The voltage-dependent steady state parameters (V_50_ and slope) of both somatic and axonal sodium channels were not significantly altered at FT as compared to PT (Figs. 2C,2D), except for the slope factor of the axonal activation currents (Suppl. Table 1–3). As a result, axonal channels showed reduced window currents (LoF change) at FT (Fig. 2D inset). Besides, somatic sodium channels displayed resistance to inactivation in response to sustained depolarization at FT (Fig. 3E,G), while axonal ones quickly underwent massive inactivation (Fig. 3F,H). This observation is consistent with the temperature responsive steady state gating properties of somatic and axonal channels in which somatic ones are around 12 mV more depolarized in the V_50_ of fast inactivation than the axonal ones at FT (Suppl. Table 1) and that elevating temperature depolarizes membrane potentials (Fig. 4B). Thus, the sustained availability of somatic ones could position them to be a key component in supporting neuronal hyper-excitabilities at FT, while the fast and massive inactivation of axonal sodium channels as a mechanism of LoF could restrict neurons from FT-induced neuronal hyper-excitabilities. Moreover, pharmacology experiments showed that inhibition of Na_V_1.2 by PTx3 could significantly suppress FT-induced enhancement of excitability in brain slice preparations (Fig. 6F,H,I). Up-regulation of Na_V_1.2 as found in *Scn8a*^*−/−*^ mice could cause an increase in neuronal excitability (Fig. 6I) and FS susceptibility in mice at FT (Fig. 7). Neuron simulations revealed weighted contributions of Na_V_1.2 and Na_V_1.6 to neuronal excitability at FT (Figs 8 and 9). Na_V_1.6 alone fails to support neuronal firing at FT, while compensatory replacement of Na_V_1.6 with Na_V_1.2 better prevent neurons from depolarization blocks (APs failure) at FT and enable neurons keep firing at FT(Figs 8J,K and 9D). These simulations provide theoretical supports to our experimental data. Thus, our data argue for an important role of Na_V_1.2 in mediating FT-induced neuronal hyper-excitability that may promote seizure onset.

The involvement of Na_V_1.2 in human disease has been highlighted by the identification of over 20 non-/mis-sense mutations in SCN2A in patients with seizure disorders, including BFNIS, GEFS+ ^5,22,25,50,51^. In a mouse model of temporal lobe epilepsy (TLE), GoF mutation in Na_V_1.2 (p.GAL879-881QQQ) which causes prolonged inactivation and increased persistent currents leads to severe spontaneous seizures and lethality within 6 months^24^. GoF mutation of Na_V_1.2 (p.A263V) which causes increased persistent currents is associated with neonatal seizures and its developmentally increasing expression in cerebellum may be accountable for the later onset of episodic ataxia^52^. Controversially, LoF mutations in SCN2A (p.R1312T) could also lead to hyperexcitability^53–57^. The exact mechanism is unknown at present. It is suggested that a reduction of Na_V_1.2 might lead to unsubdued excitatory inputs and disorganized action potential firings^53^. Noteworthily, these results were obtained from *in vitro* expression systems. Their transferability to *in vivo* situation would require further characterization. On the other hand, GoF mutations in Na_V_1.6 are associated with epileptic encephalopathy and sudden unexpected death in epilepsy (SUDEP)^28–30,32,58,59^. For example, de novo GoF mutation in SCN8A(p.N1768D) causes depolarizing shift in steady state fast inactivation, increased persistent sodium currents and non-inactivating currents, as well as increased currents in response to slow ramping depolarization^58,59^. Mice carrying this mutation recapitulate several aspects of human conditions including convulsive seizures and epileptiform EEG, and SUDEP^59^. In contrast, LoF mutations of SCN8A are associated with cognitive deficits and ataxia without seizures^27,60^. Global conditional knockout and lentivirus mediated local knockdown of Na_V_1.6 channels were shown to be efficacious for seizure control^61^. More recently, Liu and colleagues showed that transcriptional repression of SCN8A by Chromodomain Y-like (CDYL) protein suppresses epileptogenesis in mice^62^. Thus, the empirical data from different groups are supportive to our hypothesis that somatic Na_V_1.2 channels may play a key role in supporting FT-induced neuronal hyperexcitability and seizures due to their GoF changes at FT, and also to our modeling predictions regarding suppression of Na_V_1.6 as a potentially effective way (Fig. 8D, Suppl. Figure 6).

Among those properties significantly affected by FT, the role of sodium channel gating rate in excitability is worth noting. Previous work suggests that prolonged decay of sodium currents may augment sodium influx and thereby promote neuronal excitability^5,24^. Our data suggest that prolonged decay of sodium currents (correspondingly, slower gating rate) might not necessarily be a valid indication of promoting neuronal excitability. Indeed, faster gating rates (or shortened fast inactivation) were observed at higher temperature and positively correlated with enhanced excitability (Figs 3 and 4); Simulations revealed that neuronal excitability can be enhanced by decreasing sodium channel gating time constants (τ) down to 0.5 fold, a point beyond which the excitability of model neuron sharply reduces (Suppl. Figure 7A,C). Conversely, increasing gating time constants monotonically reduced firing (Suppl. Figure 7A,C). Similar results can be obtained in modeling channel gating rates directly (Suppl. Figure 7B).

As temperature exerts global effects on virtually every molecular and cellular processes, other temperature sensitive molecules, i.e., potassium channels^63,64^, TRPV^65^, calcium channels^66^ may also be affected by increased temperature. Indeed, elevated temperature causes membrane potential depolarization and input resistance reduction(Fig. 4A–D), suggesting the involvement of ion channels mediating subthreshold *V*_m_ fluctuations, such as K_V_7 potassium channels^63^, hyperpolarization-activated cyclic nucleotide gated cation channels (HCN) or sodium channels mediating persistent currents^67^. In our realistic neuron model, we took into account the contributions of the ionic currents from HCN, K_V_, Kca, Km, Ca_V_ channels which are implemented with default temperature sensitive gating mechanisms(Q_10_ = 2.3). However, the effects of specific changes at FT of these ion channels on neuronal excitability, such as expression levels, V_50_s shifts and others, are not investigated in present study due to insufficient experimental data and the scope of present study. Notably, mutation in HCN2 channels has been reported in patients with FS^68^ and was shown to undergo long lasting enhancement after FS insult which could convert potentiated synaptic inhibition to hyper-excitability^69^. Systematic investigations on the integral impacts of the different types of ion channels or receptors on neuronal excitability might reveal detail information regarding how the excitability of neurons is reshaped by elevated temperature.

Since Na_V_1.6 and Na_V_1.2 are expressed in both excitatory pyramidal neurons and inhibitory interneurons^42^, their role in regulating the changes in excitability of inhibitory neurons at febrile temperature remains to be further examined.

In summary, our data reveal differential biophysical properties and functional roles of somatic and axonal sodium channels in promoting neuronal excitability at FT, and argue that somatic sodium channels (mainly Na_V_1.2 subtype) could be a key player in mediating FT-induced seizures. These findings could provide an insightful view to clinical incidences and FS animal models in which the subjects without genetic defects may also respond to hyperthermia by seizures. Finally, investigation of temperature sensitive properties of ion channels and receptor with mutations that have been associated with febrile seizures will help to further elucidate pathophysiological mechanisms of FS in the affected pedigrees.

## Materials and Methods

### Ethics statement

All procedures involving animals followed the protocols approved by the Animal Research Advisory Committee at the Shanghai Institutes of Biological Sciences, and accorded with the guidelines for the care and use of laboratory animals approved by School of Brain and Cognitive Sciences, Beijing Normal University. All possible efforts were made to minimize the number and suffering of animals used in this study.

### Animals

To accord with a general analogy of rodent model to the first year and toddler years of human life^1^, P13–P17 SD rat or Scn8a knockout mice were used in our experiment. Data were presented as mean ± s.e.m.

### Behavior analysis of febrile seizures

For induction of febrile seizures, mice were placed in a 2 L flask in a WP-25A electrothermal incubator pre-warmed at 42.0 ± 1.0 °C and monitored for 30 min before returning to home cage. Seizure scores were based on a modified Racine scale and judged independently by two persons and discussed with third person to reach a consensus on scoring.

### Electrophysiological recording

Animals were anesthetized with 1% sodium pentobarbital before decapitation. Coronal slices from prefrontal cortex with a thickness of 300 μm were prepared as described previously^40^. We made somatic nucleated patch recordings for currents derived from Na_V_1.2 channels and isolated axonal blebs recordings for currents mainly mediated by Na_V_1.6^40,70^.

### Immunostaining

Prefrontal cortical sections at 15μm thickness were cut on cryostat for double or triple stainings for Na_V_1.2, Na_V_1.6, Na_V_1.1, Na_V_1.3, NeuN, or Ankrin G with procedures described previously^40,41^.

### Neuron modeling

To evaluate the contribution of sodium channel subtypes to neuronal excitability at FT, we performed simulations in a single compartment model and a realistic model in Neuron 7.2^71^.

### Data availability Statement

The datasets generated and/or analyzed during the current study are available from the corresponding author on reasonable request.

## Electronic supplementary material

Supplementary Information
