# Supplementary material for: Differential roles of NaV1.2 and NaV1.6 in regulating neuronal excitability at febrile temperature and distinct contributions to febrile seizures
Source: Sci Rep. 2018 Jan 15;8:753. doi: 10.1038/s41598-017-17344-8 (PMC5768682; doi:10.1038/s41598-017-17344-8)
Supplement: Supplementary file 1 — Supplementary Information [file 41598_2017_17344_MOESM1_ESM.pdf]

Supplementary Information

Differential roles of $Na_V1.2$ and $Na_V1.6$ in regulating neuronal excitability at febrile temperature and distinct contributions to febrile seizures

Mingyu Ye [2,*], Jun Yang [1], Cuiping Tian [3], Qiyu Zhu [4], Luping Yin [1], Shan Jiang [2], Mingpo Yang [2], and Yousheng Shu [1,*]

1, State Key Laboratory of Cognitive Neuroscience and Learning, School of Brain and Cognitive Sciences, the Collaborative Innovation Center for Brain Science, Beijing Normal University

2, Institute of Neuroscience, State Key Laboratory of Neuroscience, Chinese Academy of Sciences

3, iHuman Institute, ShanghaiTech University

4, Brain Institute, School of Pharmaceutical Sciences, Capital Medical University

*Correspondence should be addressed to Yousheng Shu (yousheng@bnu.edu.cn);

Postal address: 19 Xinjiekouwai St., Beijing 100875, China; Tel: 86–10–5880 4976

or Mingyu Ye (my2406@cumc.columbia.edu); present address: Department of Biochemistry and Biophysics, Columbia University Medical Center; 701 west 168th street, New York, 10032, USA..

Running title: functional dissection of sodium channel subtypes on neuronal excitability at febrile temperature

Keywords: Sodium Channel; Electrophysiology; Neuron simulation; Febrile Seizures;

MATERIALS AND METHODS

Ethics statement

All procedures involving animals followed the protocols approved by the Animal Research Advisory Committee at the Shanghai Institutes of Biological Sciences, and accorded with the guidelines for the care and use of laboratory animals approved by School of Brain and Cognitive Sciences, Beijing Normal University. All possible efforts were made to minimize the number and suffering of animals used in this study.

Animals

To accord with a general analogy of rodent model to the first year and toddler years of human life [1], age P13–P17 of both Sprague-Dawley (SD) rat and C3Fe.Cg-Scn8a$^{med/+}$J strain mice were used in this study. Initial electrophysiology studies on different sodium channel subtypes were carried out on brain slices from SD rat (Fig.1-4). C3Fe strain mice were used for behavior studies and electrophysiology (Fig. 5-7). Heterozygous C3Fe.Cg-Scn8a$^{med/+}$J mice(*Scn8a*$^{+/-}$) were from Jackson Lab (Bar Harbor, Maine, USA) and intercrossed to obtain homogzygous mice (*Scn8a*$^{-/-}$ knockout). The animals were kept on a 12h/12h light/dark cycle until use.

Electrophysiological recording

Animals were anesthetized with 1% sodium pentobarbital before decapitation. Coronal slices from prefrontal cortex with a thickness of 300 μm were cut on a Leica microtome (VT1000S) in 95% oxygenated, ice-cold slicing solution containing (in mM): 2.5 KCl, 1.25 NaH2PO4, 26 NaHCO3, 2 MgSO4, 2 CaCl2, 25 dextrose, 213 sucrose (315–325 mOsm, pH 7.2–7.3). Brain slices were continuously perfused with oxygenated external solution (see below) on an electrophysiology setup equipped with an upright infrared differential interference contrast microscope (BX51WI, Olympus) and a CCD camera (DAGE, IR-1000E) for visualization of cortical neurons.

We made somatic nucleated patch recordings for currents primarily contributed from $Na_V1.2$ channels and isolated axonal bleb recordings for currents mainly mediated by $Na_V1.6$ [2,3]. For whole-cell recordings or nucleated patch recordings, low resistance pipettes (4-6 MΩ) were made from borosilicate glass tubing using Sutter P97 (Sutter Instrument, USA). For axonal recordings, high resistance pipettes (7-9 MΩ) were used. A positive air pressure was applied before lowering the pipette into the bath solution and maintained during approaching the neurons of interest. When a dent on the surface of a neuron or an axonal bleb was formed, the air pressure was released to form Giga seal instantly. Pulses of negative air pressure was applied to form whole cell or axonal bleb recording configuration. To achieve nucleated patch recording

configuration, negative air pressure was applied and maintained after forming whole cell recording configuration to attract cell nucleus to the pipette tip. Patch pipette was gently retracted out from the brain tissue. Giga seal was maintained during the whole process of making a nucleated patch. The access resistance was less than 20 MΩ for whole cell recordings, and around 25 MΩ for axonal bleb recordings and nucleated patch recordings.

The patch pipettes were filled with intracellular solution containing (in mM) 140 CsCl, 2 $MgCl_2$, 2 Na_2ATP, 10 HEPES, 10 EGTA (287 mOsm, pH 7.2 with CsOH). The normal ACSF contained (in mM) 125 NaCl, 2.5 KCl, 2 $MgSO_4$, 2 $CaCl_2$, 26 $NaHCO_3$, 1.25 NaH_2PO_4 and 25 dextrose (315 mOsm, pH 7.4). To obtain purely Na^+ currents, we added 20 TEA, 3 4-AP and 0.1 $CdCl_2$ in the external solution to block the K^+ and Ca^{2+} currents. In current clamp experiments, intracellular solution contains (in mM) 140 KGluconate, 3 KCl, 2 $MgCl_2$, 10 HEPES, 0.2 EGTA, and 2 Na_2ATP; the extracellular solution was normal ACSF. Alexa Fluor 488 (100 μM) was added to the pipette solution to visualize the recorded neurons.

The protocols for voltage-dependent activation/inactivation, kinetics of recovery from inactivation and onset inactivation of sodium channels are referred to suppl. Fig. 2, 3. The normalized *G-V* curves were averaged and fitted by a Boltzmann equation,

$$\frac{G}{G_{max}} = \frac{1}{1+e^{\frac{V_{50}-V}{K}}} \quad \text{(Equation(Eq.) 1)}$$

where *G* is the conductance, *Gmax* is the maximal conductance, V_{50} is the voltage at which sodium channels are half activated or inactivated, and *K* is the slope factor. The time constants were determined by single-exponential fits.

Data were low-pass filtered at 10 kHz and sampled at 100 kHz using MultiClamp 700B and Digidata 1440A with pClamp 10.2 (Molecular Devices, CA, USA) or Micro1401 with Spike2 software (CED, Cambridge, UK). Leak currents were online subtracted by a P/4 protocol. Only those recordings with resting leak currents no greater than -80 pA were used for data analysis. All presented membrane potentials (V_m) in voltage clamp recordings were not corrected for liquid junction potentials (2.5 mV). Temperature of brain slices were monitored by a closely placed thermistor and controlleVmd by a dual automatic temperature controller (TC-344B, Warner Instr. Inc.).

Immunostaining

A detailed procedure for immunostaining was described previously[2,4,5]. Briefly, prefrontal cortical brain tissues were cut into 15 μm thick sections on cryostat. Slices were incubated in a blocking solution (5% normal goat serum, 0.3% Triton X-100 in PBS) at room temperature for 2 h, followed by overnight incubation in primary antibodies at 4 ℃. The primary antibodies used in this study are mouse anti-$Na_V1.1$ (73-023, NeuroMab;1:200), rabbit anti-$Na_V1.2$ (ASC-002, Alomone Labs;1:400), mouse anti-$Na_V1.2$ (73-024, NeuroMab; 1:200), rabbit anti-$Na_V1.3$ (ASC-004, Alomone Labs; 1:200), rabbit anti-$Na_V1.6$ (ASC-009, Alomone Labs; 1:400), mouse anti-PV (MAB1572, Millipore; 1:1000), goat anti-Ankyrin G (sc-31778, Santa cruz; 1:400), mouse anti-AnkG (sc-12719, Santa cruz; 1:400), mouse anti-NeuN (MAB377, 1:1000; Millipore). After thoroughly washed in PBS, slices were further incubated in secondary antibodies (1:1,000; Invitrogen) for 2 h at room temperature. The 2nd antibodies are Alexa 488–conjugated donkey anti-rabbit, Alexa 555–conjugated donkey anti-mouse, or Alexa 647–conjugated donkey anti-goat. Brain slices were finally washed in PBS and mounted with fluoromount-G (Electron microscopy science). Images were taken on a laser scanning confocal microscope (Nikon FN1) with 20X, or 60x objective.

3D-reconstruction of neuron morphology

Pyramidal neurons filled with 0.2% Biocytin during whole cell recording were stained by Avidin-Biotin – Peroxidase method, and visualized with 3,3'-diaminobenzidine (DAB) according to manufacture protocols (SK-4100, Vector Labs). Neurons were imaged with 1 μm z-stack on Neurolucida workstation (MBF Bioscience, IL, USA) equipped with Nikon Eclipse E600FN microscopy and a Plan Apo 40X/0.95 DIC objective lens. The neurites of a typical pyramidal neuron were traced with Simple Neurite Tracer in FIJI software. Morphology reconstruction was then processed in Neurostudio for 3D-coordinates of neurites and in Neurolucida for multiple soma contours along z stacks. The resulting ASC coordinates were converted to HOC codes in Neuron7.2 for modeling.

Neuron modeling

To evaluate the contribution of sodium channel subtypes to neuronal excitability at FT, we constructed a single compartment model and a realistic model using Neuron 7.2[6]. Single compartment model (10 μm diameter, 190.98 μm length, 1 compartment, 6000 μm^2 surface area) was implanted with $Na_V1.2$ (gbar_na12 = 20 $pS/\mu m^2$), $Na_V1.6$ (gbar_na16 = 50 $pS/\mu m^2$), K_V (gbar_K_V = 30 $pS/\mu m^2$) channels and a passive conductance (g_pas = 1 $pS/\mu m^2$). The membrane capacitance is 0.75 $\mu F/cm^2$. For evaluating the

components of sodium conductance underlying backpropagating APs, AP waveforms experimentally recorded at different temperatures were assigned to an expanded SEClamp6 to measure the underlying sodium channel subtypes' conductance. Trapezoidal numerical integration method was used to calculate the area of sodium conductance. To evaluate contributions of different sodium channel subtypes to neuronal excitability at different temperatures, we designed channel subtype replacement experiments. The initial density of each sodium channel subtype was set at 200 pS/μm^2. The total sodium channel density was kept constant. To simulate knockout of one channel subtype with a compensatory upregulation of the other channel subtype, if the density of one channel subtype was set to 0 pS/μm^2, the other one was correspondingly increased to 400 pS/μm^2. To evaluate the effect of channel gating time constants or gating rate on neuronal excitability, we set temperature at 36.5 °C, then alter the state variable time constants (τ_m, τ_h) or channel open/close rate constants.

In the realistic neuron model, we applied a 3-D reconstructed morphology of an experimentally recorded layer 5 pyramidal neuron. The model neuron contains 143 sections and 322 compartments in total and has surface area of 65,607 μm^2. The soma comprises 54 z-stack contours and has an area of 2,323 μm^2. Axon hillock with length of 12.4 μm tapered from 7.0 to 3.0 μm is connected to the soma, which is followed by an axon initial segment (49.7 μm in length) and then unmyelinated axon segment (183.4 μm). Immediately followed is an axon bleb (diameter: 4.4 μm) and then an axon branch (148.8 μm). We also attached an artificial 1000-μm myelinated axon separated by 10 nodes of Ranvier (1 μm). The apical and basal dendrites comprise 71 and 46 branches with total length of 5, 107.0 μm and 2, 532.8 μm, and total area of 40, 346.8 μm^2 and 16, 741.3 μm^2, respectively. We modeled spiny dendrites based on published simple spine density model [7]. The passive membrane properties, Cm (membrane capacitance), Rm (membrane resistance), Ra (axial resistivity), are set to 0.75 μF/cm^2, 30 kΩ cm^2, 125 Ω cm for dendrites, soma, hillock, AIS and unmyelinated axon. The Cm and Rm are set to 0.02 μF/cm^2 and 30 kΩ cm^2 for myelinated axon, and 0.75 μF/cm^2 and 75 Ω cm^2 for bleb and Ranvier nodes.

The density distribution mechanisms of ion channels were based on previous models and immunostaining data [2,7,8]. Briefly, $Na_V1.2$ and $Na_V1.6$, as well as potassium channels (K_V, Km, K_{Ca}), high voltage activated calcium channels (I_{Ca}) and HCN channels (I_h) were implanted in the realistic model. Transient $Na_V1.2$ channels present at soma with 80 pS/μm^2 and at hillock with 48 pS/μm^2. Channel density was non-uniform along the AIS, with the highest density (3,072 pS/μm^2) at approximately 22 ~ 32 μm from soma. Transient $Na_V1.6$ currents are presented in the AIS (peak density of 1, 920 pS/μm^2 at about 72 μm from soma), nakeaxon (1,200 pS/μm^2), bleb (1,200 pS/μm^2) and nodes (1,600 pS/μm^2). The myelinated axon contains low-density panNa currents (20 pS/μm^2). The dendrites are implanted with panNa (80 pS/μm^2),

Kv (10 pS/μm^2), Km (0.3 pS/μm^2), K_{Ca} (3 pS/μm^2) and Ca channels (0.3 pS/μm^2). The non-inactivating Kv channels are also presented in soma (20 pS/μm^2), hillock (100 pS/μm^2), AIS (linearly increased with distance to a maximum value of 1, 000 pS/μm^2), nakeaxon (150 pS/μm^2), bleb (150 pS/μm^2) and nodes (250 pS/μm^2). An internal calcium concentration mechanism is only implanted in somatodendritic region. HCN channels have an exponential distribution from dendrites to the soma, and are absent in the axon [8].

Ion channel mechanisms of Na_V1.2 and Na_V1.6 were revised from previous models[7]. Sodium currents (I_{Na}) were described by:

$$I_{Na} = tadj \cdot g_{Na} \cdot m^3 \cdot h \cdot (V - E_{Na}) \quad \text{(Eq. 2)}$$

where g_{Na} is the local sodium conductance density, m and h are the activation and inactivation state dependent variables, V is the local Vm, E_{Na} is the reversal potential for Na^+ ion (E_{Na} = 70 mV, measured: 70.6 ± 1.3 mV), tadj is a temperature dependent variable based on Q_{10}. The state variables (m, h) are given by the following formula:

$$\alpha_{m\_Na12} = 0.3 \cdot \frac{V+19}{1-e^{-\left(\frac{V+19}{6.9}\right)}} \quad \text{(Eq.3)}$$

$$\alpha_{m\_Na16} = 0.32 \cdot \frac{V+32}{1-e^{-\left(\frac{V+32}{5.45}\right)}} \quad \text{(Eq.4)}$$

$$\beta_{m\_Na12} = 0.08 \cdot \frac{(19-V)}{1-e^{\left(\frac{V-19}{6.9}\right)}} \quad \text{(Eq.5)}$$

$$\beta_{m\_Na16} = 0.12 \cdot \frac{(32-V)}{1-e^{\left(\frac{V-32}{5.45}\right)}} \quad \text{(Eq.6)}$$

$$\alpha_{h\_Na12} = 0.02 \cdot \frac{V+50}{1-e^{-\left(\frac{V+50}{7.6}\right)}} \quad \text{(Eq.7)}$$

$$\alpha_{h\_Na16} = 0.024 \cdot \frac{V+60}{1-e^{-\left(\frac{V+60}{6.6}\right)}} \quad \text{(Eq.8)}$$

$$\beta_{h\_Na12} = 0.0091 \cdot \frac{-(V+60)}{1-e^{\left(\frac{V+60}{7.6}\right)}} \quad \text{(Eq.9)}$$

$$\beta_{h\_Na16} = 0.0091 \cdot \frac{-(V+75)}{1-e^{\left(\frac{V+75}{6.6}\right)}} \quad \text{(Eq.10)}$$

$$h_{\infty\_Na12} = \frac{1}{1+e^{\left(\frac{V+56.2}{7.6}\right)}} \quad \text{(Eq.11)}$$

$$h_{\infty\_Na16} = \frac{1}{1+e^{\left(\frac{V+67.8}{6.6}\right)}} \quad \text{(Eq.12)}$$

$$\tau_m = \frac{1 \times Var1}{tadj \times (\alpha_m + \beta_m)} \quad \text{(Eq.13)}$$

$$\tau_h = \frac{1 \times Var1}{tadj \times (\alpha_h + \beta_h)} \quad \text{(Eq.14)}$$

$$m_\infty = \frac{\alpha_m}{\alpha_m + \beta_m} \quad \text{(Eq.15)}$$

$$\text{tadj} = Q_{10}^{\left(\frac{Celsius-36.5}{10}\right)} \quad \text{(Eq.16)}$$

Ionic mechanisms for voltage-gated non-inactivating potassium channels (K_V), muscarinic activated slow non-inactivating potassium channels (K_M), calcium-dependent potassium channels (K_{Ca}), voltage-dependence HCN channels and high-voltage activated calcium channels were adopted from published models [7,8]. The integration time step in our simulation is 0.025 ms. Based on our data, the Q_{10}s for current

amplitudes of $Na_V1.2$ and $Na_V1.6$ were set as 2.04 and 1.50, respectively, if temperature is higher than PT; and 1.36 and 1.16 when temperature lower than PT.

Behavior analysis of febrile seizures

Mice were placed in a 2L flask in a WP-25A electrothermal incubator (Taisite Instrument Co., LTD, Tianjin, China) prewarmed at 42.5 ± 1.0 ℃ for 30 min before returning to home cage. Both the core (rectal) and ambient temperatures were monitored in real-time. Seizure responses were videoed and scored based on a modified Racine scales which include 5 stages.

Stage 1. Hypoactivity: reduced motion, hypotonia, staring, licking, grooming, hunched posture or crawling.
Stage 2. Partial seizures: forelimb extension while lying on its belly, trembling at the head, autonomic signs including salivation, hyperventilation.
Stage 3. Clonic seizure: occasional focal clonus affecting mostly the head and/or forelimbs(s), raring, wild or circular walking/running, jumping, loss of balance and control.
Stage 4. Status epilepticus: loss of upright posture, severe clonic-tonic convulsions while lying on its side or belly. Jerking. Complete loss of balance and control.
Stage 5. Death

Seizure scores were judged independently by two persons and discussed with third person to reach a consensus on scoring. We adopted the following formula [9] for evaluating seizure severity.

$$\text{Seizure Severity} = \frac{\sum \text{scores of a given mouse}}{\text{time of experiment}} \quad \text{(Eq. 17)}$$

FS temperature threshold and latency refer to the point when the mice developed stage 4 phenotypes that can be unambiguously defined.

Statistic Analysis

Data were processed in GraphPad Prism5.0 (GraphPad Software Inc. San Diego, CA) and Matlab 2011b (MathWorks, Natick, MA) software. Data were presented as mean ± s.e.m., and compared by one-way ANOVA with post-hoc Bonferroni test or student t-test as indicated. The n value represents the number of neurons from at least three mice. The *p* values less than 0.05 were considered statistically significant.

Suppl. Figures 1-5

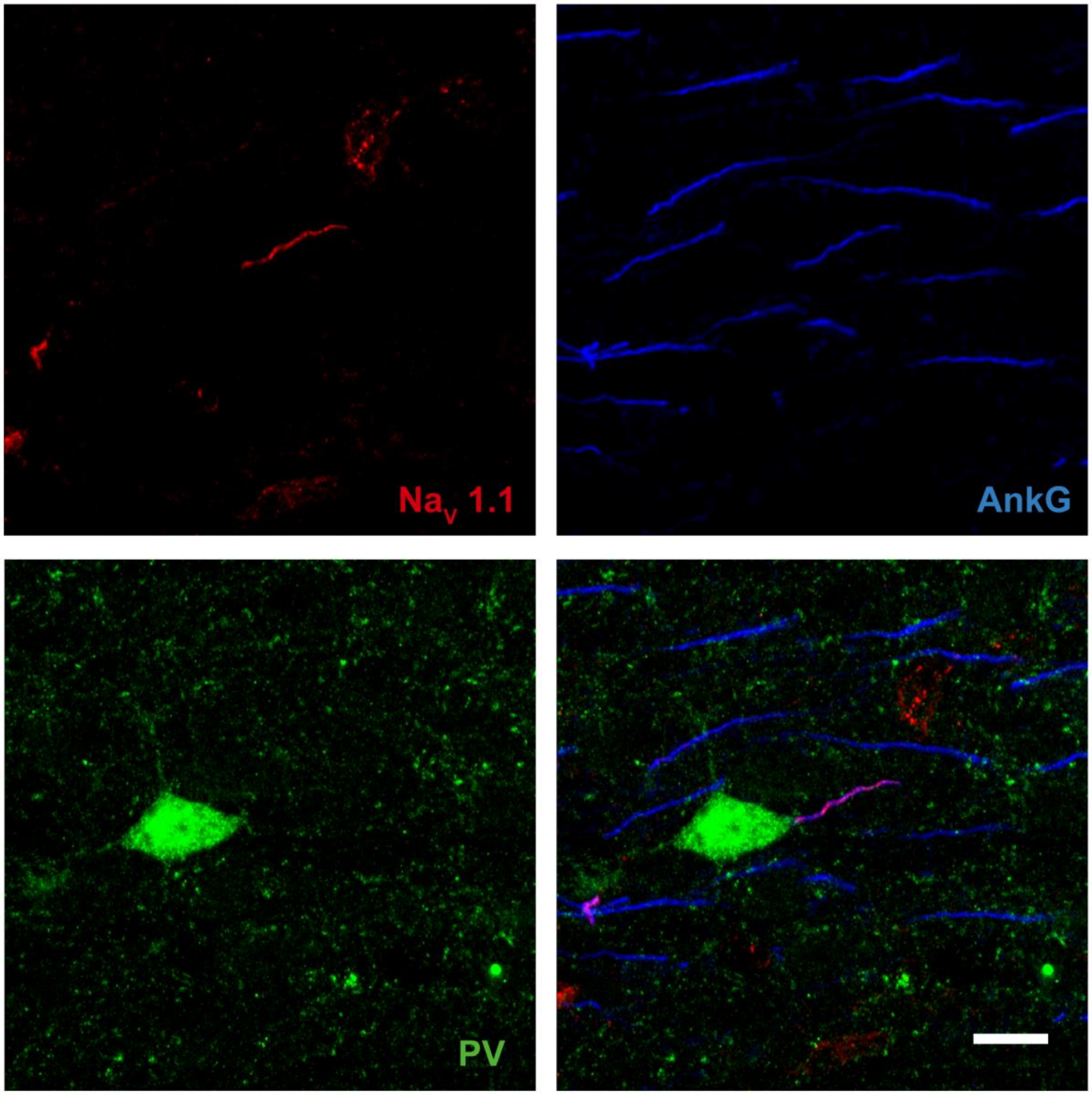

Suppl. Fig. 1 Na$_V$1.1 is expressed on PV+ interneurons but not putative excitatory cells in prefrontal cortical tissues. Triple immunostaining for Na$_V$1.1(red), PV(green), AnkG(blue); Scale bar: 10 µm.

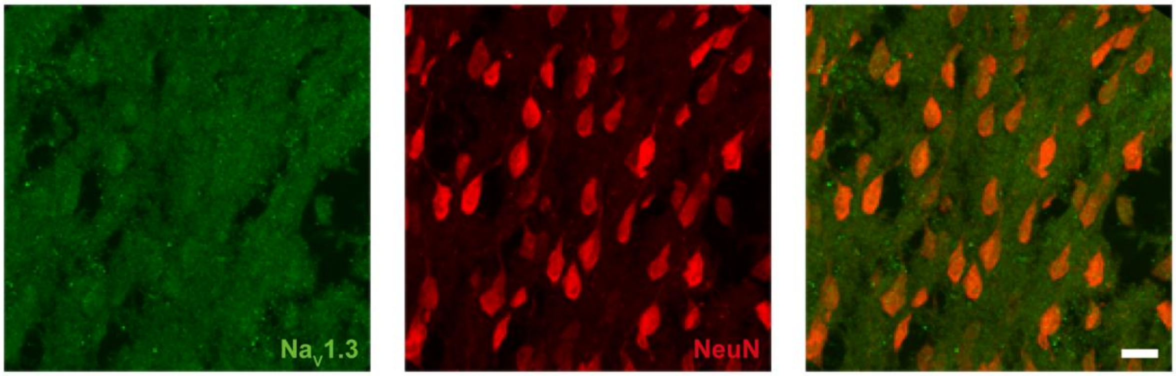

Suppl. Fig. 2 Na$_V$1.3 is undetectable on postnatal 15 prefrontal cortical tissues. Double immunostainning for Na$_V$1.3(green), NeuN(red; neuronal marker); Scale bar: 20 µm.

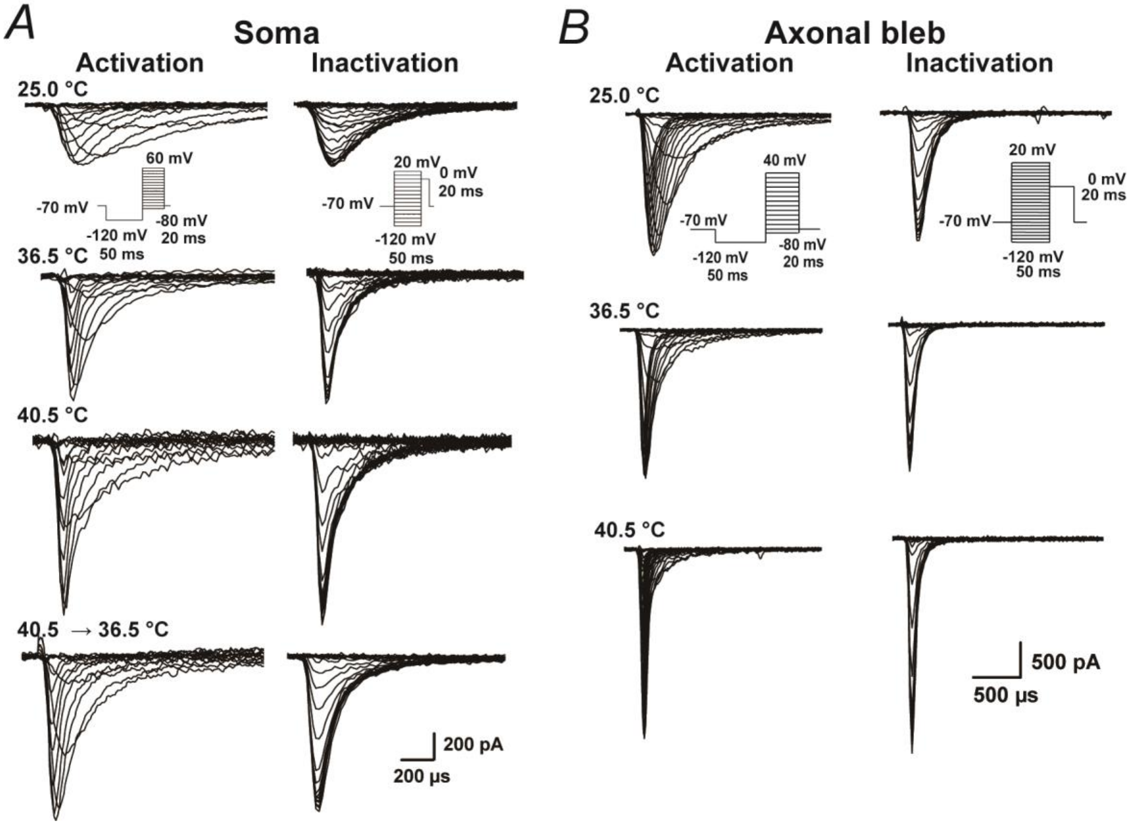

Suppl. Fig. 3 (A) Representative somatic sodium channels' activation and inactivation traces at different temperatures. Insets illustrated corresponding voltage clamp protocols. (B) Similar to (A) but for sodium channels expressed on axon-trunk.

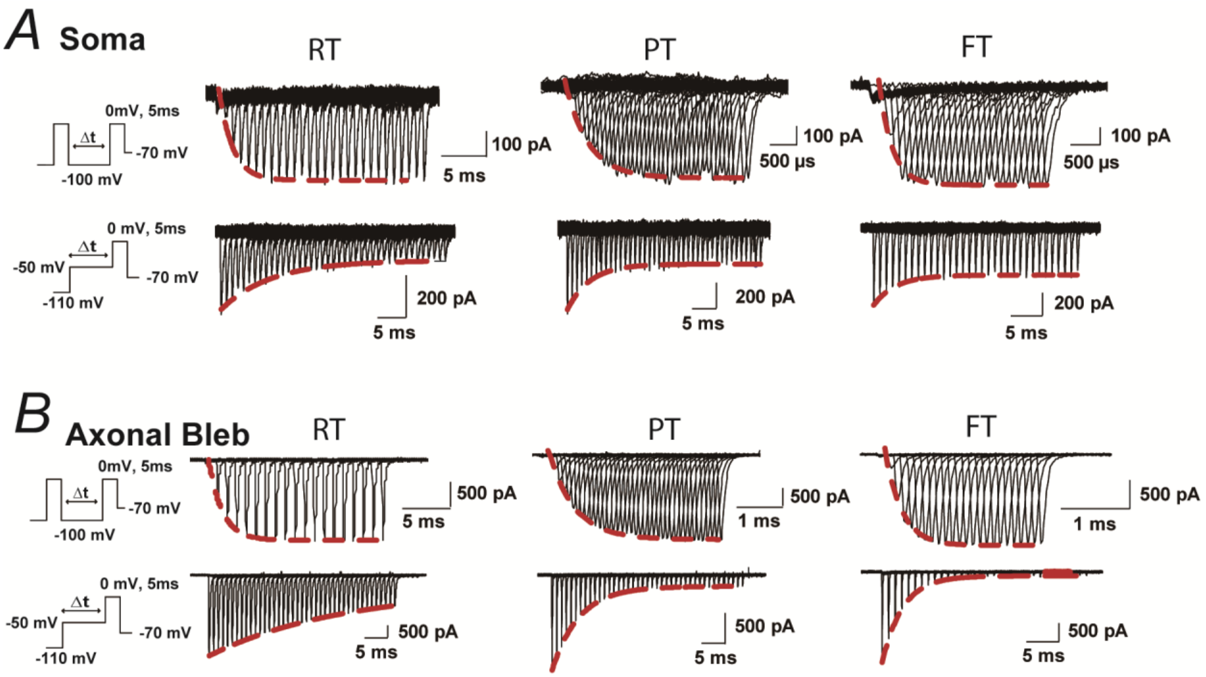

Suppl. Fig. 4 (A) Representative traces showing the kinetics of recovery from inactivation (upper row) and onset inactivation (bottom row) for somatic sodium channels at different temperature. Insets showed corresponding voltage clamp protocols. (B) Similar to (A) but for axon-trunk sodium channels. Red dash lines show exponential fits to peak sodium currents.

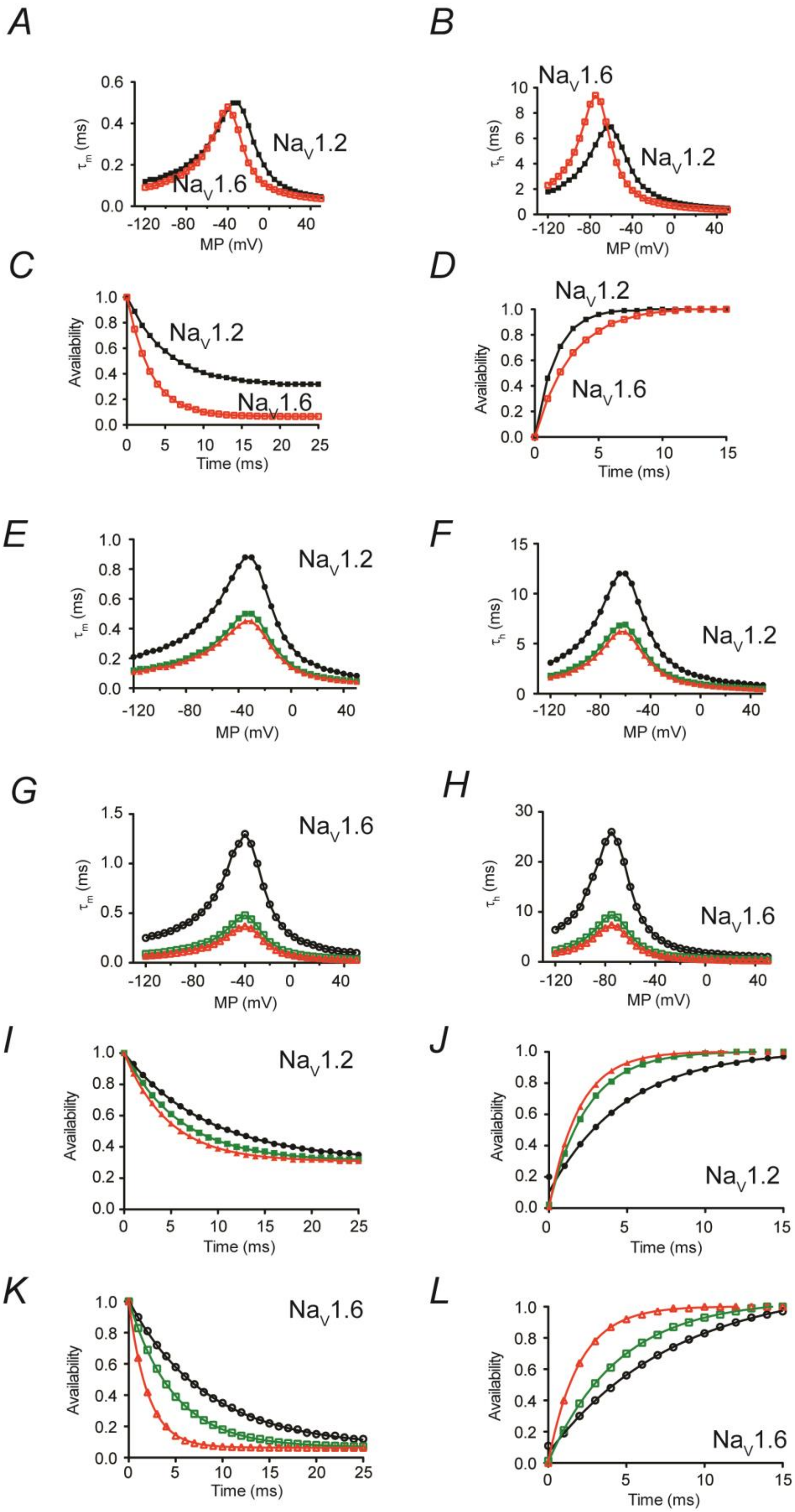
A
B
C
D
E
F
G
H
I
J
K
L
Na$_V$1.2
Na$_V$1.6
τ_m (ms)
τ_h (ms)
MP (mV)
Availability
Time (ms)

Suppl. Fig. 5 Simulations on the gating properties and temperature responsive properties of Na_V1.2 and Na_V1.6 channels in single compartment model. Comparisons of channel open (τm, A) and inactivation($\tau_{h,}$ B) gating rates as a function of membrane potential at PT. (C-D) Comparisons of the kinetics of onset inactivation (C) and the kinetics of recovery from inactivation (D) between Na_V1.2 (black traces) and Na_V1.6 (red traces) subtypes at PT. (E-F)simulations on the effects of temperature on voltage dependent τ_m (E) and τ_h (F) of Na_V1.2. Black curves:RT; Green: PT; Red: FT. Similar color code applies to the following sub-figures. (G-H): similar as (E-F), but for Na_V1.6. (I-J) simulations on the effects of temperature on the kinetics of onset inactivation of Na_V1.2. (K-L) similar as (I-J), but for Na_V1.6.

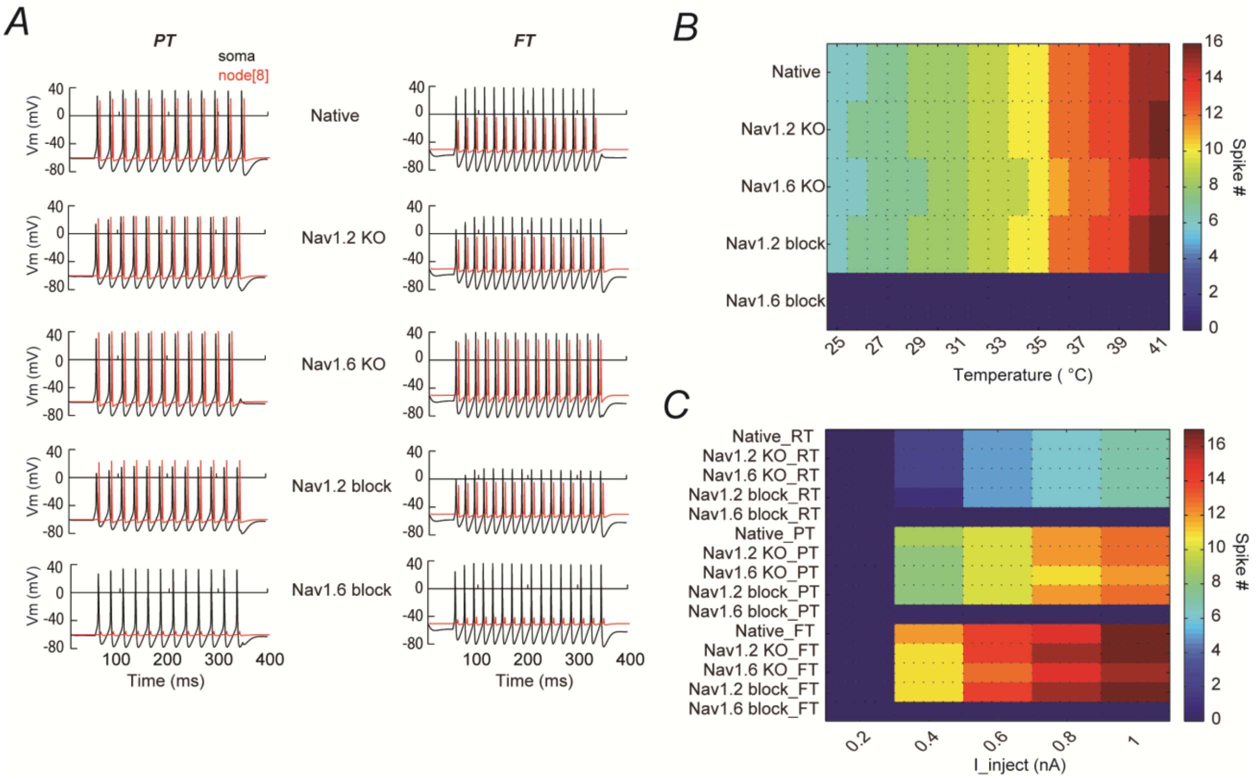

Suppl. Fig. 6 Simulation of AP firing rates and AP propagation velocity in realistic neuron model. (A) Firing patterns of the realistic model neuron at PT (left column) and FT (right column) with different operations on sodium channel subtypes. Red traces: APs at the 8th node of Ranvier. Black traces: APs at the soma. (B-C): Heat map illustration on the consequences of different sodium channel subtype manipulations on AP firing rates at the 8th node across a temperature range from 25 ℃ to 41 ℃ (B) and a series of current intensities from 0.2 nA to 1 nA (C). Note that blockage of $Na_V1.6$ conductance effectively abolishes AP propagation.

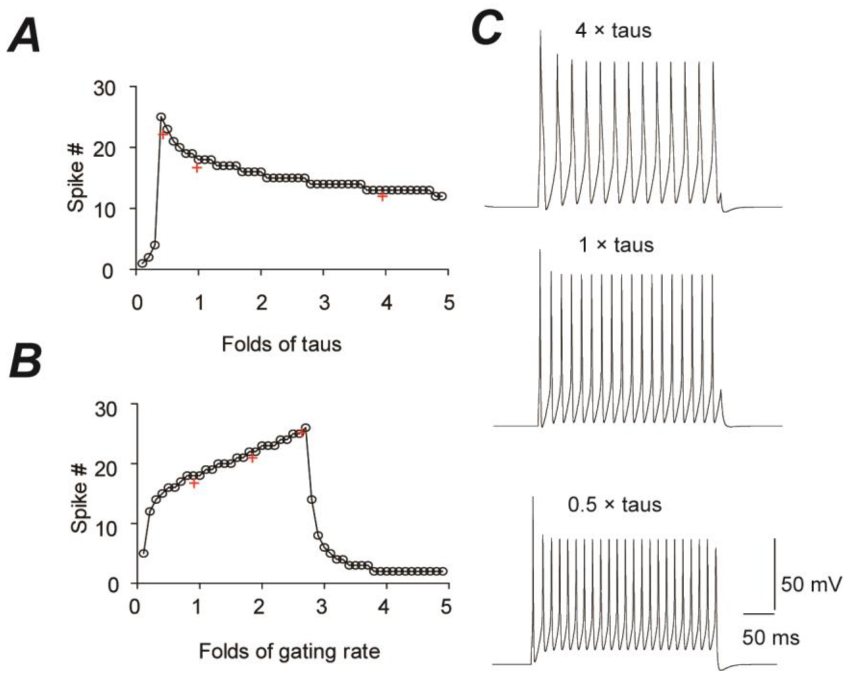

Suppl. Fig. 7 Simulation on the effect of sodium channel gating time constants (A) or gating rate (B) on neuronal excitability. (C) Representative firing pattern of the model neuron at 0.5x, 1x and 4x time constants of sodium channel subtypes. Red crosses indicate 0.5x, 1x and 4x taus in (A), and 1x, 2x, 2.7x gating rates in (B).

Suppl. Table 1 Properties of somatic and axonal sodium channels at different temperatures

	Soma				Axonal bleb		
	RT	PT	FT	FT →PT	RT	PT	FT
**Amp.(pA)**	-422.1±17.1	-604.1±33.4	-803.3±70.3	-820.4±74.3	-2.1±0.2	-2.5±0.2	-3.0±0.3
**τ_ decay(ms)[&]**	0.32±0.01	0.19±0.01	0.15±0.01	0.13±0.01	0.168±0.005	0.069±0.002	0.056±0.002
**τ_recov. (ms)**	2.21±0.47	0.58±0.05	0.30±0.02	N.D.	1.45±0.14	0.69±0.07	0.28±0.05
**τ_dev. (ms)**	14.3±2.2	5.4±0.5	4.9±1.1	N.D.	15.6±1.8	6.5±1.3	2.9±0.2
**V_{50}_Act. (mV)**	-19.5±0.6	-18.9±0.9	-16.4±0.7	-18.4±1.3	-26.7±1.0	-29.1±1.0	-28.6±1.1
**k_Act.**	6.8±0.2	6.4±0.2	6.0±0.2	6.4±0.3	5.7±0.2	4.1±0.2	3.2±0.3
**V_{50}_Inact (mV)**	-61.2±0.8	-56.3±0.8	-54.4±1.1	-57.9±1.1	-68.1±1.1	-66.3±1.2	-65.4±1.0
**k_Inact**	-8.3±0.2	-6.6±0.1	-6.2±0.2	-6.3±0.2	-5.9±0.2	-5.5±0.1	-5.6±0.2

#: Unit: nA; &: measured at 0mV. N.D.: not determined.

Suppl.Table2 Statistics for the gating properties of somatic sodium channels between different temperatures

	Amp.	V_{50}_act	V_{50}_inac	k_act	k_inact	τ_ decay[&]	τ_recov.	τ_dev.
RT vs PT	*	NS	**	NS	***	***	***	**
RT vs FT	***	NS	***	*	***	***	***	**
RT vs FTPT	***	NS	NS	NS	***	***	ND	ND
PT vs FT	*	NS	NS	NS	NS	**	##	NS
PT vs FTPT	**	NS	NS	NS	NS	***	ND	ND
FT vs FTPT	NS	NS	NS	NS	NS	NS	ND	ND

&: measured at 0mV. *: p<0.05; **: p<0.01; ***:p<0.0001, 1-way ANOVA with Bonferroni's Multiple Comparison Test; ##: p<0.01; two-tailed unpaired t-test. NS: no significant; ND: no determined.

Suppl.Table3 Statistics for the gating properties of somatic sodium channels between different temperatures

	Amp.	V_{50}_act	V_{50}_inac	k_act	k_inact	τ_ decay[&]	τ_recov.	τ_dev.
RT vs PT	NS	NS	NS	***	NS	***	***	***
RT vs FT	**	NS	NS	***	NS	***	***	***
PT vs FT	NS	NS	NS	*	NS	###	**	#

&: measured at 0mV. *: p<0.05; ***:p<0.0001, 1-way ANOVA with Bonferroni's Multiple Comparison Test; #: p<0.05, ###: p<0.001, two-tailed unpaired t-test. NS: no significant;

Suppl. Table 4 p-values of paired t-test for the comparisons of spike numbers between PT and FT in Fig. 5E-H

Current (X100pA)	WT	WT_PTx3	KO	KO_PTx3
1	NA	NA	0.3656	0.1801
2	0.004	0.1362	0.377	0.2778
3	0.0004	0.0141	0.381	0.6434
4	0.0035	0.0557	0.013	0.6593
5	0.1397	0.0621	0	0.1329
6	0.9515	0.5119	0	0.0177
7	0.0009	0.8523	0	0.0004
8	0	0.9634	0	0
9	0	0.4884	0	0
10	0	0.2478	0.0001	0.0017
11	0	0.1715	0.0005	0.0153
12	0	0.0127	0.0109	0.0117
13	0	0.4111	0.0097	0.1972
14	0	0.0028	0.0578	0.529
15	0	0.0677	0.0551	0.2002
16	0	0.321	0.0113	0.5907
17	0	0.187	0.0046	0.4483
18	0	0.229	0.025	0.4237
19	0.0002	0.2535	0.1098	0.2766
20	0.0007	0.2734	0.0677	0.6768

Reference

1 Reid, A. Y., Galic, M. A., Teskey, G. C. & Pittman, Q. J. Febrile seizures: current views and investigations. *Can J Neurol Sci* **36**, 679-686 (2009).

2 Hu, W. *et al.* Distinct contributions of Na(v)1.6 and Na(v)1.2 in action potential initiation and backpropagation. *Nat Neurosci* **12**, 996-1002, doi:10.1038/nn.2359 (2009).

3 Hu, W. & Shu, Y. Axonal bleb recording. *Neurosci Bull* **28**, 342-350 (2012).

4 Li, T. *et al.* Action potential initiation in neocortical inhibitory interneurons. *PLoS Biol* **12**, e1001944, doi:10.1371/journal.pbio.1001944 (2014).

5 Tian, C., Wang, K., Ke, W., Guo, H. & Shu, Y. Molecular identity of axonal sodium channels in human cortical pyramidal cells. *Front Cell Neurosci* **8**, 297, doi:10.3389/fncel.2014.00297 (2014).

6 Carnevale, N. T. & Hines, M. L. *The NEURON book*. (Cambridge University Press, 2006).

7 Mainen, Z. F. & Sejnowski, T. J. Influence of dendritic structure on firing pattern in model neocortical neurons. *Nature* **382**, 363-366 (1996).

8 Kole, M. H., Hallermann, S. & Stuart, G. J. Single Ih channels in pyramidal neuron dendrites: properties, distribution, and impact on action potential output. *J Neurosci* **26**, 1677-1687, doi:26/6/1677 (2006).

9 Gimenez-Cassina, A. *et al.* BAD-dependent regulation of fuel metabolism and K(ATP) channel activity confers resistance to epileptic seizures. *Neuron* **74**, 719-730, doi:S0896-6273(12)00343-1 (2012).
